# Scalable neutral H_2_O_2_ electrosynthesis by platinum diphosphide nanocrystals by regulating oxygen reduction reaction pathways

**DOI:** 10.1038/s41467-020-17584-9

**Published:** 2020-08-06

**Authors:** Hui Li, Peng Wen, Dominique S. Itanze, Zachary D. Hood, Shiba Adhikari, Chang Lu, Xiao Ma, Chaochao Dun, Lin Jiang, David L. Carroll, Yejun Qiu, Scott M. Geyer

**Affiliations:** 1 Department of Chemistry, Wake Forest University, Winston-Salem, NC 27106 USA; 2 Shenzhen Engineering Lab of Flexible Transparent Conductive Films, School of Materials Science and Engineering, Harbin Institute of Technology, Shenzhen, 518055 China; 3 Center for Nanophase Materials Sciences (CNMS), Oak Ridge National Laboratory (ORNL), Oak Ridge, TN 37831 USA; 4 Department of Materials Science and Engineering, Massachusetts Institute of Technology, Cambridge, MA 02139 USA; 5 Material Science and Technology Division (MSTD), Oak Ridge National Laboratory (ORNL), Oak Ridge, TN 37831 USA; 6 Center for Nanotechnology and Molecular Materials, Department of Physics, Wake Forest University, Winston-Salem, NC 27109 USA; 7 Institute of Functional Nano and Soft Materials (FUNSOM), Soochow University, Suzhou, Jiangsu 215123 China

**Keywords:** Catalyst synthesis, Energy, Electrocatalysis

## Abstract

Despite progress in small scale electrocatalytic production of hydrogen peroxide (H_2_O_2_) using a rotating ring-disk electrode, further work is needed to develop a non-toxic, selective, and stable O_2_-to-H_2_O_2_ electrocatalyst for realizing continuous on-site production of neutral hydrogen peroxide. We report ultrasmall and monodisperse colloidal PtP_2_ nanocrystals that achieve H_2_O_2_ production at near zero-overpotential with near unity H_2_O_2_ selectivity at 0.27 V vs. RHE. Density functional theory calculations indicate that P promotes hydrogenation of OOH* to H_2_O_2_ by weakening the Pt-OOH* bond and suppressing the dissociative OOH* to O* pathway. Atomic layer deposition of Al_2_O_3_ prevents NC aggregation and enables application in a polymer electrolyte membrane fuel cell (PEMFC) with a maximum r(H_2_O_2_) of 2.26 mmol h^−1^ cm^−2^ and a current efficiency of 78.8% even at a high current density of 150 mA cm^−2^. Catalyst stability enables an accumulated neutral H_2_O_2_ concentration in 600 mL of 3.0 wt% (pH = 6.6).

## Abstract (web summary)

The synthesis of high concentration H_2_O_2_ from water and oxygen at moderate conditions could provide an on-site H_2_O_2_ source for medical and water purification applications. Here, authors show Al_2_O_3_-stabilized PtP_2_ nanocrystals to enable selective, stable and efficient neutral pH H_2_O_2_ production.

## Introduction

Hydrogen peroxide (H_2_O_2_) is a valuable chemical for a variety of industrial applications, as well as a potential energy carrier alternative to oil or hydrogen in fuel cells. H_2_O_2_ is currently manufactured by a large-scale indirect anthraquinone process and the under-developed direct synthesis from a H_2_ and O_2_ mixture^1^. The anthraquinone process involves multiple redox reaction steps and requires expensive palladium-based hydrogenation catalysts. Furthermore, energy-intensive distillation for obtaining high concentration H_2_O_2_ is necessary to minimize transportation and storage costs. Direct synthesis via H_2_ and O_2_ is more straightforward but potentially explosive. Electrochemical H_2_O_2_ production through the oxygen reduction reaction (ORR) in an electrolyzer or fuel cell is an attractive and cost-effective route due to its mild operation conditions, on-site production, and tunable concentration^2^. However, it is still a great challenge to develop efficient and stable electrocatalysts that are selective toward the two-electron ORR.

Incorporation of an efficient and stable catalyst into a proton-exchange membrane electrolyzer or fuel cell is a promising route to commercialization. Many potential ORR electrocatalysts, mainly including carbon-based materials and noble metal-based materials, have been reported for H_2_O_2_ production in alkaline or acidic electrolyte^3^. Carbon-based electrocatalysts typically perform well in alkaline solution but show low intrinsic activity and stability in acidic media^4^. Bimetallic noble metal alloys, such as Au–Pd, Pt–Hg, Pd–Hg, and Au–Pt–Ni, catalyze ORR through two-electron pathways with selectivity as high as 95%^5–7^. The goal of secondary metal incorporation is to change the electronic structure of the primary catalytic site and optimize the binding strength of reaction intermediates^8^. An ideal two-electron ORR electrocatalyst should possess a suitable binding strength for OOH* (not too strong or too weak) and suppress the O–O bond breakage in OOH* to O*. However, stability of the bimetallic alloys is a concern, particularly for medicinal or water treatment applications. Elemental leaching hinders the long-term ORR stability and the leached ingredient (particularly toxic Hg) devalues the H_2_O_2_ product and increases the separation cost^9,10^. Apart from the metal alloying strategy, incorporation of non-metal elements such as phosphorus, sulfur, and boron into metals to form multicomponent alloys has been demonstrated to be an attractive and effective way to improve the electrocatalytic activity of metal catalysts^11–15^. Particularly, our previous work found that electronegative phosphorus (P) was able to regulate the binding strength of intermediates in ORR and improve the four-electron ORR activity for cobalt phosphide^16^. Since Pt is the most widely used material for four-electron ORR, it is highly desirable to investigate if P alloying is able to alter the electronic structure of P-rich platinum phosphide and shift the reaction pathway from a four-electron to a two-electron pathway.

Ultrasmall nanoparticle catalysts not only have a high surface to volume ratio which reduces cost for precious metal catalysts by increasing the per mass surface area, but also lead to higher exposure of low-coordinated edge sites for improved electrocatalytic activity^17^. However, small nanoparticle electrocatalysts are prone to aggregation during long-term electrochemical operation, which lowers surface area, reduces available active sites and consequently causes electrocatalytic activity degradation^18^. Encapsulation of nanoparticles in a porous ultrathin shell of a stable metal oxide can largely preserve the catalytic activity while increasing the resistance against aggregation^19^. Atomic layer deposition (ALD), a thin-film deposition technique that allows growth of conformal coating through a self-limiting vapor growth process, has been employed to deposit ultrathin metal oxide layers to overcoat and stabilize nanoparticle catalysts for optimizing both activity and durability^20–22^. Therefore, ALD is of interest to prevent aggregation of ultrasmall platinum phosphide nanocrystals and preserve their ORR activity and selectivity during long-term electrocatalysis.

Generally, the ORR activity of an electrocatalyst is initially evaluated by the rotating-ring disk electrode (RRDE) technique, which provides only an upper limit to the activity and selectivity of two-electron ORR due to rapid transportation of H_2_O_2_ from disk to ring^2^. In real devices, such as those based on a membrane electrode assembly (MEA) architecture, additional transport factors must be considered. These include the slow diffusion rate of H_2_O_2_ from the catalyst and gas diffusion layers to the output stream and the chance for further H_2_O_2_ reduction or chemical decomposition^23^. Yamanaka and Wilkinson’s groups have reported MEA-based water electrolyzers and fuel cell reactors for production of small amounts of neutral H_2_O_2_ by continuously feeding gaseous O_2_ into the cathodic chamber^24–27^. However, the long-term stability for efficient O_2_-to-H_2_O_2_ production at high current levels is still a large challenge due to the severe H_2_O_2_ accumulation at the interface between ORR catalyst layer and proton-exchange membrane, particularly if there is no solvent flow to remove the concentrated product. To reach a high concentration at a large scale will require incorporation of an efficient two-electron ORR catalyst into a system with sufficient long-term stability in the presence of high H_2_O_2_ content to allow for continuous recycling of the H_2_O_2_ product to reach medical level concentrations.

Herein, monodisperse colloidal platinum diphosphide nanocrystals (PtP_2_ NCs) with an uniform size of 3 nm are directly synthesized by a hot-injection method using platinum(II) 2,4-pentanedionate and tris(trimethylsilyl)phosphine. Unlike ORR catalyzed by Pt NCs which follow a conventional four-electron pathway, the ultrasmall PtP_2_ NCs proceed through a two-electron pathway with a nearly zero overpotential to initialize the O_2_-to-H_2_O_2_ reaction and achieve a maximum selectivity of 98.5% at 0.27 V vs. RHE. DFT calculations reveal that changes in electron density and increased Pt atom separation due to P incorporation leads to a weaker adsorption of the key OOH* intermediate and inhibition of subsequent O–O breakage of OOH* to form the O* intermediate. The ultrasmall PtP_2_ NCs are treated by ALD of an alumina overcoat and post-annealing to suppress aggregation and maintain electrocatalytic stability. The resulting catalyst is employed in a PEMFC to achieve a steady neutral H_2_O_2_ formation rate of 2.26 mmol h^−1^ cm^−2^ and the accumulated H_2_O_2_ concentration reaches 3 wt% in 65 h and as high as 1.21 M in 600 mL after continuous cycling for 120 h.

## Results

### Synthesis and characterization

**Fig. 1:**
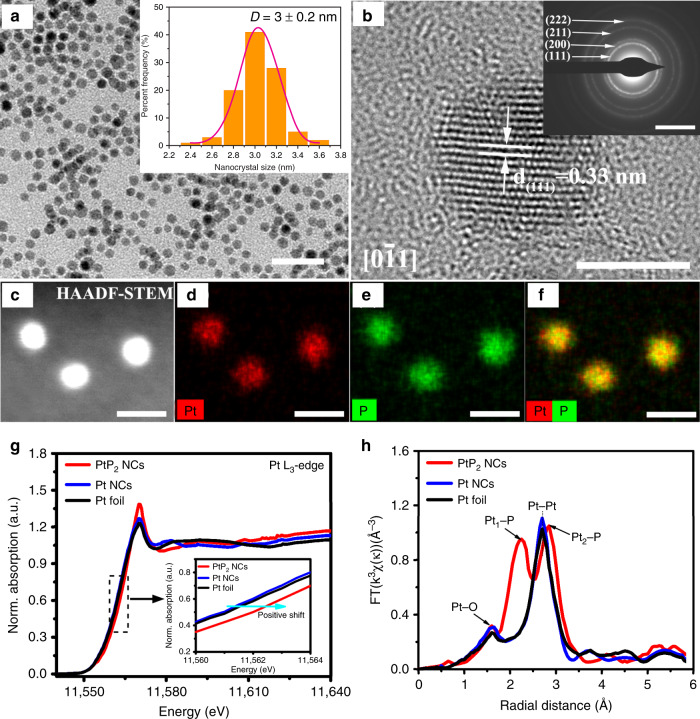
Materials characterization of PtP_2_ NCs. **a** TEM image; scale bar, 20 nm. **b** HRTEM image; scale bar, 2 nm, inset is the selected-area electron diffraction (SAED) image; scale bar, 5 1/nm. **c** HAADF-STEM, and **d**–**f** elemental mapping images of PtP_2_ NCs; scale bar, 5 nm. **g** Pt L_3_-edge X-ray absorption near-edge structure (XANES) and **h** extended X-ray absorption fine structure (EXAFS) spectra of PtP_2_ NCs, Pt NCs, and Pt foil.

To synthesize ultrasmall and highly monodisperse platinum phosphide NCs, platinum(II) 2,4-pentanedionate and tris(trimethylsilyl)phosphine ((Me_3_Si)_3_P) are employed in a hot-injection synthesis to allow for separate control of the nucleation and growth processes. In a typical preparation, 0.3 mmol of platinum(II) 2,4-pentanedionate (0.118 g) was initially mixed with 8 mL oleylamine (OAm), 0.5 mL oleic acid (OA), and 8 mL octadecene (ODE). Oxygen and impurities were removed by placing the solution under vacuum at 120 °C for 1 h. In a nitrogen atmosphere, the solution was then heated to 220 °C at a rate of 10 °C/min. Meanwhile, the P precursor was prepared by placing 1.2 mL (Me_3_Si)_3_P dissolved in hexane (10 wt%) and 1.0 mL ODE under vacuum to remove the hexane at room temperature. The (Me_3_Si)_3_P solution was quickly injected at 220 °C and for 15 min the temperature was maintained. Inductively coupled plasma mass spectroscopy (ICP–MS) gives an atomic ratio of platinum to phosphorus of 0.498 (Supplementary Table 1), and energy-dispersive X-ray spectroscopy (EDS) of platinum phosphide NCs shows a similar Pt:P ratio of 0.509 (Supplementary Fig. 1), confirming the formation of PtP_2_. The XRD pattern of PtP_2_ NCs matches the cubic structure of bulk PtP_2_ (ICDD PDF: 01-080-2220) (Supplementary Fig. 2). The central Pt atoms are surrounded by 6 phosphorus atoms, which are situated at the corners of a slightly distorted octahedron. Similarly, the phosphorus atoms are surrounded by one phosphorus and three platinum neighbors (Supplementary Fig. 3). The transmission electron microscopy (TEM) image of the as-synthesized PtP_2_ NCs shows a spherical morphology with average size of 3 ± 0.2 nm (Fig. 1a). A well-resolved lattice fringe with interplane distances of 0.33 nm is observed, corresponding to the (111) crystallographic plane of the cubic PtP_2_ (Fig. 1b). The (111) plane, associated with other planes, such as (200), (211), and (222), are shown on the corresponding selected-area electron diffraction (SAED) image (inset of Fig. 1b), indicating good crystallinity of the as-synthesized PtP_2_ NCs. The well-defined spherical geometry and high monodispersity of the PtP_2_ NCs is clearly observed from the high-angle annular dark-field scanning TEM (HAADF-STEM) image (Fig. 1c). The elemental mapping of the PtP_2_ NCs shows that the Pt and P elements are evenly distributed throughout the whole nanocrystal region (Fig. 1d–f).

X-ray absorption spectroscopy was carried out to reveal the local geometric and electronic structures of PtP_2_ and Pt NCs. Figure 1g shows the Pt L_3_-edge X-ray absorption near-edge structure (XANES) spectra of PtP_2_ NCs, Pt NCs, and Pt foil. The rising edge of PtP_2_ NCs shows a positive shift compared with that of Pt NCs and Pt foil due to the increased valence oxidation state after incorporation of P into Pt. This result can be ascribed to an electron density shift from metallic Pt to local P atoms with high electronegativity. The donor-acceptor nature of electron density distribution in PtP_2_ NCs is further confirmed by the X-ray photoelectron spectroscopy (XPS) of Pt 4f (Supplementary Fig. 5). The binding energy of Pt 4f_7/2_ for PtP_2_ NCs is positively shifted (0.9 eV) from that of Pt 4f_7/2_ for Pt NCs. This shift is larger than that of conventional metal alloying, suggesting that the stronger electron delocalization in PtP_2_ NCs may have greater influence on the intrinsic electronic properties and reaction intermediates adsorption/desorption behavior^28^. The intensity of the Pt L_3_ white line is a qualitative indicator of electron vacancies in the 5d orbitals of Pt atoms. The white line intensity for PtP_2_ NCs is higher than that for Pt NCs and Pt foil, which is attributed to an electron density shift from Pt to P which creates electron vacancies in Pt and increases probability for electron transition from 2p to the unoccupied 5d orbital^29^. The Fourier transforms of the k^3^-weighted extended Pt L_3_-edge X-ray absorption fine structure (EXAFS) spectra were shown in Fig. 1h. The EXAFS fitting results are summarized in Supplementary Fig. 6 and Table 2. The small peak located at 1.60 Å is assigned to the typical Pt–O bond which is derived from trace amounts of surface platinum oxide. The peaks at 2.25 and 2.85 Å are both attributed to the Pt–P bond, suggests diverse P coordination to the central Pt. Interestingly, no fitting peak is observed within 3.00 Å for the Pt-Pt bond in PtP_2_, which is consistent with an expected Pt-Pt distance in the range of 3.6–4.2 Å in the PtP_2_ cubic crystal structure. This length difference has significant influence on the intermediate adsorption on the platinum bridge site, which will be discussed in detail later.

### Electrochemical ORR performance

**Fig. 2:**
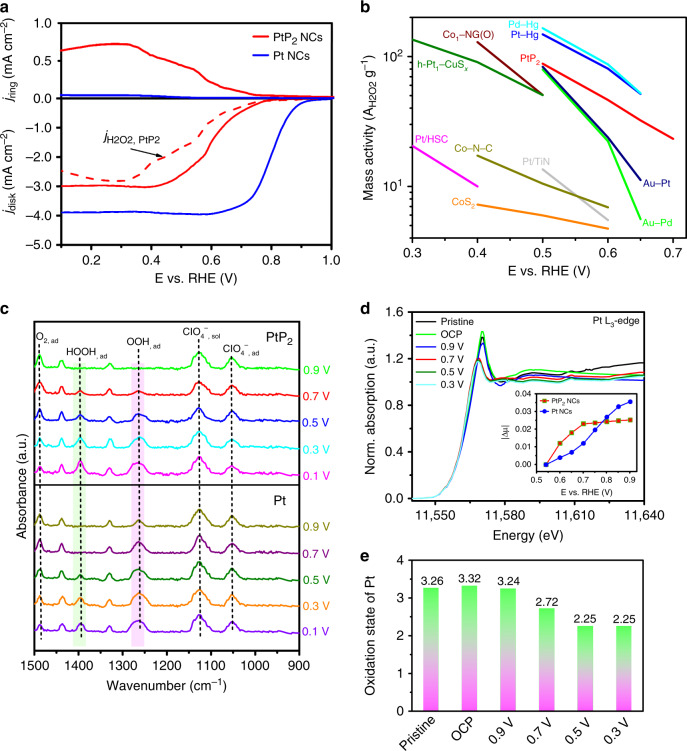
Electrochemical and in situ characterization of PtP_2_ NCs. **a** RRDE voltammograms at 1600 rpm in O_2_-saturated electrolyte with the disk current density, ring current density, and current density corresponding to hydrogen peroxide obtained from the ring current. **b** Mass activity of different electrocatalysts for H_2_O_2_ production in acidic electrolyte. **c** In situ ATR-IR spectra and **d** in situ Pt L_3_-edge XANES spectra collected on the PtP_2_ electrodes at constant potential in O_2_-saturated 0.1 M HClO_4_. Inset in **d** shows the impact of potential on the Pt L_3_-edge XANES spectra: Δ*μ* = *μ*(*V*) − *μ*(0.54 V). **e** Pt oxidation state in PtP_2_ as a functional of applied constant potential.

The ORR was performed in O_2_-saturated 0.1 M HClO_4_ solution using a RRDE. The potential of the ring is set such that it can oxidize the H_2_O_2_ produced at the disk electrode, with the resulting current providing a measurement of the level of H_2_O_2_. For the disk current, the PtP_2_ NCs show an onset potential (defined as the potential at which a current density of 0.1 mA/cm^2^ is achieved) of 0.716 V vs. RHE, which is significantly shifted from that of Pt NCs (0.88 V vs. RHE) (Fig. 2a). A remarkable ring current in the potential range of 0.1–0.708 V vs. RHE is observed on PtP_2_ NCs, compared to a negligible current measured for the Pt NCs control. The slightly higher onset potential of the PtP_2_ NCs than the thermodynamic limit of 0.70 V vs. RHE could potentially be ascribed to a Nernst-related potential shift. The PtP_2_ NCs achieve a maximum H_2_O_2_ selectivity of 98.5% at 0.27 V vs. RHE, and the corresponding electron transfer number (*n*) is 2.03 (Supplementary Fig. 8), while the Pt NCs follow a typical four-electron pathway during ORR process. This indicates that phosphorus incorporation can regulate the electronic structure of surface Pt active sites and therefore alter the ORR pathway. Figure 2b summarizes the mass activity of different electrocatalysts for O_2_-to-H_2_O_2_ conversion, and further details of these calculations are available in Supplementary Table 3. The remarkable mass activity and low overpotential of PtP_2_ is superior to most reported electrocatalysts. While it is inferior to the state-of-the-art Pt–Hg and Pd–Hg alloys, replacement of Hg with minimal loss in efficiency is promising since the potential for Hg to leach directly into a medicinal product is a considerable barrier for practical application.

To directly compare stability, highly monodispersed Pt–Hg nanocrystals were also synthesized (Supplementary Fig. 9). The PtP_2_ NCs show very similar mass activity to the-state-of-art Pt-Hg NCs (Supplementary Fig. 10), but the leaching of heavy metals is greatly reduced as determined by ICP–MS. The concentration of Hg leached from Pt–Hg NCs is 74.6 × 10^3^ ppb after ORR for 6 h in 0.1 M HClO_4_, which is three orders of magnitude higher than the Pt and P leached from PtP_2_ NCs (Supplementary Table 4). This severe leaching phenomenon limits the wide application of H_2_O_2_ produced by the Pt–Hg nanoparticle electrocatalyst, particularly for medicine or water purification. In general, the chemical stability of bimetallic Pt–Hg alloy catalysts is highly dependent on their synthesis routes and surface properties and further studies need to be done to systematically establish Hg leaching discrepancy between the discussed Pt–Hg NCs and the reported electrodeposited Pt–Hg nanoparticles^6^.

In situ attenuated total reflection infrared spectroscopy (ATR-IR) was carried out to investigate adsorbed oxygen intermediates on the supported PtP_2_ catalyst during ORR in O_2_-saturated 0.1 M HClO_4_ (Fig. 2c). As detailed below, the in situ observation of OOH_ad_ and HOOH_ad_ during ORR confirms the associative two-electron pathway for PtP_2_. In accordance with previous literature, the bands at 1435 and 1330 cm^−1^ are assigned to the functional groups of the carbon support, and the bands at 1126 and 1052 cm^−1^ are ascribed to the ClO_4_^−^ species^30^. The absorption band at 1488 cm^−1^, present at all applied potentials, is assigned to the O–O stretching mode of adsorbed molecular oxygen (O_2,ad_). This assignment agrees with previous report that the band of adsorbed O_2_ is observed around 1468 cm^−1^ for Pt/C^31^. The band intensity shows a continuous decrease with increase of overpotential due to O_2_ consumption as well as substitution by the newly formed oxygen species (Supplementary Fig. 11a). This band disappears in N_2_-saturated solution and shifts to 1406 cm^−1^ in the ^18^O_2_ isotope spectrum (Supplementary Fig. 12). The band at 1264 cm^−1^ can be assigned to the O–O stretching mode of adsorbed superoxide (OOH_ad_), which is consistent with that reported for the Au electrode^32^. It appears at 0.7 V vs. RHE and increases with increasingly negative potential (Supplementary Fig. 11b), which is consistent with the trend of the H_2_O_2_ current in the linear sweep voltammograms of PtP_2_ NCs. The OOH_ad_ band shows a significant shift to 1193 cm^−1^ in ^18^O_2_ (Supplementary Fig. 10)_,_ while remaining the same in the deuterated medium (Supplementary Fig. 13). As for Pt NCs, the OOH_ad_ band is initially observed at 0.9 V vs. RHE, consistent with its onset potential of four-electron ORR. Lastly, the band at 1396 cm^−1^ is attributed to the OOH bending mode of adsorbed hydroperoxide (HOOH_ad_)^33^. A slight shift is observed for HOOH_ad_ in ^18^O_2_ (Supplementary Fig. 12), while the band is not detected in the deuterated medium presumably due to a large shift to lower wavenumber (Supplementary Fig. 13). The HOOH_ad_ band first appears at 0.7 V vs. RHE for PtP_2_ NCs, in accordance with the onset in H_2_O_2_ production.

Figure 2d shows the normalized in situ Pt L_3_-XANES spectra of PtP_2_ NCs recorded at various ORR potentials in 0.1 M HClO_4_. Commercial PtO_2_, PtCl_2_, and Pt foil were used as references and a linear combination of XANES spectra was fitted to the in situ Pt L_3_-edge spectra (Supplementary Figs. 14 and 15). The calculated oxidation states of platinum species in PtP_2_ under various potentials are shown in Fig. 2e and summarized in Supplementary Table 5. For the as-prepared PtP_2_ NCs, the initial oxidation state of platinum species is calculated to be +3.26, which is attributed to the electron density shift from Pt to P. Under open-circuit potential (OCP) and 0.9 V, a slight change of oxidation state is observed. This is presumably attributed to the formation and dissolution of minor surface oxide based on the small change of white line intensity. At a potential of 0.7 V a negative shift in the absorption edge is observed the average oxidation state decreases to +2.72 since the surface platinum active sites in PtP_2_ start to be involved in O_2_ activation and H_2_O_2_ generation. The oxidation state further decreases to +2.25 by 0.5 V and remains the same to 0.3 V. It should be noted that the oxidation state of +2.25 keeps stable at 0.3 V for 6 h of measurement (Supplementary Fig. 16), indicating that the high P content can stabilize Pt^+2.25^ in the PtP_2_ for sustaining rapid and stable O_2_-to-H_2_O_2_ conversion.

The inset of Fig. 2d illustrates the changes in the Pt L_3_-edge XANES spectra with the in situ potential. Here, Δ*μ* is the value obtained by subtracting the Pt L_3_-edge XANES spectrum at 0.54 V from that collected at elevated potentials. The |Δ*μ*| of Pt NCs increase monotonically with potential, while for PtP_2_ |Δ*μ*| increases sharply from 0.54–0.7 V but remains unchanged to 0.9 V. The value of |Δ*μ*| is expected to increase with increasing amounts of oxygen species adsorption^34^. The relatively high |Δ*μ*| for PtP_2_ within 0.54–0.7 V indicates a high coverage of oxygen intermediates in this region of intermediate activity, potentially due a rate limiting step beyond generation of the initial oxygen intermediates such as *OOH adsorption.

### Theoretical DFT calculation for ORR

**Fig. 3:**
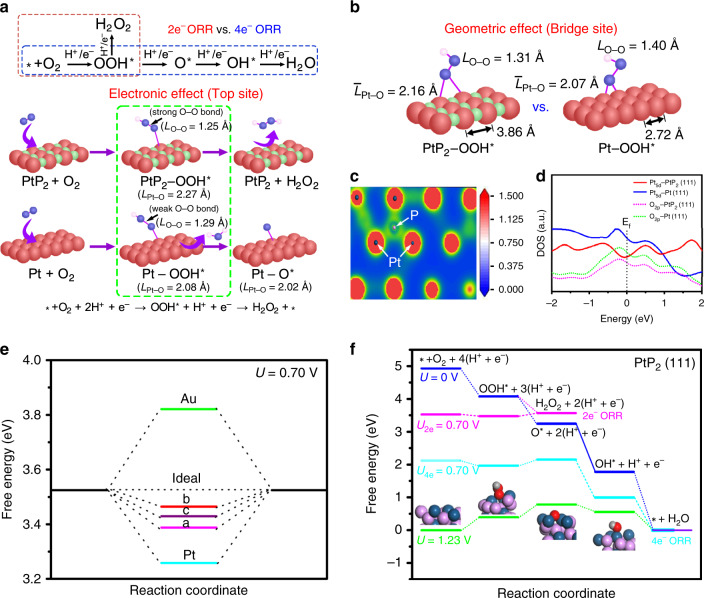
DFT analysis of reaction intermediates. **a** Key oxygen intermediates for PtP_2_ and Pt during two-electron and four-electron ORR pathways. OOH* adsorbed on top site of PtP_2_ and Pt is compared. **b** Difference between adsorption behavior of OOH* on bridge site of PtP_2_ and Pt. **c** Bader charge distribution of PtP_2_. **d** Partial density of states (PDOS) for PtP_2_ (111) and Pt (111) with adsorbed OOH*. **e** Free-energy diagram for O_2_-to-H_2_O_2_ at 0.70 V. **f** Free-energy diagram for the two-electron and four-electron ORR on PtP_2_.

Two-electron ORR generally follows two hydrogenation steps for H_2_O_2_ production, i.e., O_2_ → OOH* → H_2_O_2_, while four-electron ORR proceeds with four successive hydrogenation steps for H_2_O generation, i.e., O_2_ → OOH* → O* → OH* → H_2_O (Fig. 3a). The optimized geometries of OOH*, O*, and OH* intermediates on PtP_2_ (111) are shown in Supplementary Fig. 17. Recent theoretical studies have pointed out that the ORR activity for H_2_O_2_ production is strongly related to the binding ability of OOH*^6^. Specifically, for an ideal catalyst for H_2_O_2_ production, the adsorption of OOH* should be neither too strong nor too weak.

The adsorption configuration of OOH* is determined by the orientation type of the adsorbed oxygen molecule. There are usually two different adsorption configurations, end-on adsorption and side-on adsorption, for O_2_ on the catalytic surface of Pt and PtP_2_ (Supplementary Fig. 18). For pure Pt, the optimized adsorption energy (*E*_ads_) of the end-on configuration is more negative than that of side-on configuration, indicating that the former orientation is more favorable and subsequently that associative hydrogenation prevails over the dissociative reaction pathway. For PtP_2_, the adsorption energy of the end-on configuration is three times that of side-on configuration, suggests that the side-on adsorption of O_2_ is less favored to occur on the PtP_2_ surface and therefore the end-on adsorption of OOH* become the dominating configuration. Moreover, the optimized adsorption configuration of O* on PtP_2_ (111) is only available on top and bridge sites since simulations of O* adsorbed on hollow sites yielded bridge sites following optimization (Supplementary Fig. 17). By contrast, the O* adsorbed on hollow site on Pt (111) is more stable than that on the top and bridge sites because of strong back electron donation from Pt to O orbitals in the triple bonding state. However, the O_2_ molecule and OOH* intermediate adsorbed on hollow site of Pt (111) surface are less favorable, which leads to higher energy barrier for the conversion of OOH* to O* at the hollow site. Figure 3e depicts the free-energy diagram for two-electron O_2_-to-H_2_O_2_ pathway at equilibrium potential of 0.7 V vs. RHE. The OOH* on the typical Au and Pt metal catalysts is too weak and too strong, respectively. The free energy of OOH* (G_OOH*_) on top (a), bridge (b), and hollow (c) sites of PtP_2_ (111) is 3.39, 3.47, and 3.43 eV, respectively. Compared to Pt, the average free-energy difference compared to the ideal value of 3.52 eV is 0.09 eV, which is less than half that for Pt (0.261 eV). The weaker adsorption of OOH* on PtP_2_ (111) can be explained by both electronic and geometric effects resulting from incorporation of P. For the top site of PtP_2_ (111), the bond length of Pt–O in PtP_2_–OOH* is 2.27 Å, longer than that in Pt–OOH* (2.08 Å) (Fig. 3a). This effect is attributed to electron delocalization in phosphorus-rich PtP_2_ (Fig. 3c). For the bridge site of PtP_2_ (111), the average Pt–O bond length is increased because the Pt–Pt distance is larger due to the high degree of P incorporation (Fig. 3b).

A lower overlap among the binding states between Pt_5d_ and O_2p_ is observed when OOH* is adsorbed on PtP_2_ (111) compared with Pt (111) (Fig. 3d), leading to a weaker binding strength of OOH* over PtP_2_ (111) surface. Figure 3f shows the potential-dependent free-energy diagram for two-electron and four-electron ORR pathways on the PtP_2_ (111) surface. At zero electrode potential (*U* = 0 V) all elementary steps of the ORR are exothermic. At the equilibrium potential (*U* = 1.23 V), based on the free energies of the intermediates species the first two steps are predicted to be rate determining for the four-electron ORR. At an electrode potential of 0.7 V, the free-energy difference between OOH* to H_2_O_2_ is 0.106 eV compared to 0.180 eV for that of OOH* to O*, consistent with the experimental observation that the adsorbed OOH* preferentially undergoes hydrogenation to form H_2_O_2_ rather than continuing the dissociation path under a small overpotential. This result can be understood not only by the weakening of the Pt–OOH* bond, but also by the tightening of the O–O bond in OOH* adsorbed to PtP_2_ relative to *OOH adsorbed on Pt. For both the top and bridge sites on PtP_2_, the O–O bond length of adsorbed OOH* is shorter than that on Pt, which is beneficial to suppress the OOH*-to-O* dissociation tendency.

### ALD overcoat for stabilization of ultrasmall PtP_2_ NCs

**Fig. 4:**
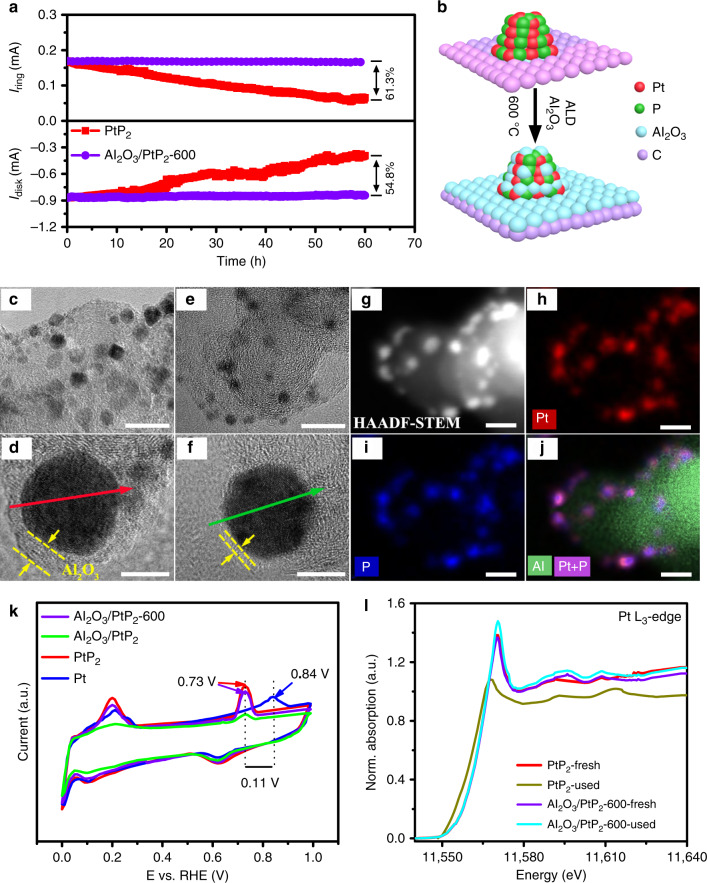
Stabilization of NCs by ALD of Al_2_O_3_. **a** Disk and ring current stability of PtP_2_ and Al_2_O_3_/PtP_2_-600 measured at a constant potential of 0.4 V vs. RHE for 60 h. **b** Depiction of Al_2_O_3_ coating by ALD and subsequent activation. TEM images for **c**, **d** Al_2_O_3_/PtP_2_ and **e**, **f** Al_2_O_3_/PtP_2_-600; scale bar, **c**, **e** 20 nm; **d**, **f** 3 nm. **g**–**j** HAADF-STEM image and corresponding elemental mapping for Al_2_O_3_/PtP_2_-600; scale bar, 10 nm. **k** Electrochemical CO stripping tests and **l** Pt L_3_-edge XANES spectra of as-prepared samples before and after ORR.

Although the ultrasmall PtP_2_ NCs gave efficient and selective O_2_-to-H_2_O_2_ conversion, both the disk and ring current quickly decayed and around 60% activity was lost within 60 h at an applied potential of 0.4 V vs. RHE (Fig. 4a). Considering that the elemental composition, crystalline structure, and surface electronic structure of PtP_2_ all remain the same following the stability test (Supplementary Fig. 19), the degradation is ascribed to size instability, which leads to nanocrystal aggregation (Supplementary Fig. 20) and a significant decrease in the electrochemical active surface area (EASA, Supplementary Fig. 21). The left shift of Pt L_3_-rising edge of XANES is also attributed to small size nanocrystal aggregation (Fig. 4l). Ultrathin metal oxides by ALD have been applied to encapsulate the supported nanoparticles and improve the size stability of nanoparticle catalysts^35,36^. In order to minimize additional electrocatalytic effects from ALD overcoating layer, we chose the relatively inert Al_2_O_3_ rather than the typical transition metal oxide promotors (e.g., Co_3_O_4_^37^, MnO^38^, Fe_2_O_3_^39^, and MoO_3_^40^). ALD of a thin Al_2_O_3_ layer was carried out to stabilize the ultrasmall PtP_2_ NCs on a commercial carbon support by alternately exposing the sample to cycles of trimethylaluminum (TMA) and water at 175 °C (Fig. 4b). Figure 4c shows that the average size of PtP_2_ NCs is increased to 5.2 ± 0.4 nm due to minor thermal aggregation during the mild-ALD process. The Al_2_O_3_ overcoat thickness is 1.8 ± 0.2 nm after 42 cycles (Fig. 4d, Supplementary Fig. 22a). This overcoat thickness is smaller than that expected for the typical ALD growth rate for Al_2_O_3_ of 1.19 Å cycle^−1^, ascribed to the site blocking from any hydrophobic organic ligands remaining on the PtP_2_ NCs surface (Supplementary Fig. 23). We determined that 42 cycles of ALD Al_2_O_3_ is required to stabilize PtP_2_ NCs and maintain ORR durability, but without further processing the ring current shows a 32.5% decrease compared to the uncoated sample (Supplementary Fig. 24). An observed decrease in EASA is consistent with an expected blocking of active sites by the ALD layer (Supplementary Fig. 25). A balance between increasing the stability and maintaining high activity is achieved by annealing the sample following ALD at 600 °C in N_2_ gas for 2 h. The size dispersity and element distribution are well maintained (Fig. 4e, g–i), while the overcoat thickness is diminished (~0.54 nm) (Fig. 4f, Supplementary Fig. 22b) and the EASA and Brunauer–Emmett–Teller (BET) surface area is almost restored (Supplementary Fig. 25). The disk and ring current of the annealed sample (Al_2_O_3_/PtP_2_–600) remain the same before and after 60 h of ORR testing in 0.1 M HClO_4_ (Fig. 4a), indicating that the Al_2_O_3_ overcoat and activation can stabilize the PtP_2_ NCs, while maintaining open pathways to the NC surface (Supplementary Fig. 26). It should be noted that the O_2_-to-H_2_O_2_ selectivity is nearly the same after the ALD surface modification, indicating that the ultrathin Al_2_O_3_ overcoating has a negligible geometric effect on the electrocatalytic selectivity of PtP_2_ electrode (Supplementary Fig. 27). The Pt L_3_-edge XANES and corresponding EXAFS of PtP_2_, Al_2_O_3_/PtP_2_, and Al_2_O_3_/PtP_2_-600 were compared in Supplementary Fig. 28. No change in the absorption edge (*E*_0_) and Pt–P bond distance was observed, which suggests that the intrinsic electronic structures of PtP_2_ were well maintained after ALD coating and annealing treatment. This could be further supported by the Pt 4f XPS results (Supplementary Fig. 29). The slight increase for the white line intensity of XANES attributes to the formation of small amount of platinum oxide during ALD process. To further evaluate the effect of the activated coating on the electrochemical properties, CO desorption (Fig. 4k) was studied by electrochemical CO stripping. Prior to the activation step, the shift in the oxidation peak is lost after Al_2_O_3_ overcoating and the current decreases suggesting the active surface is blocked. We also note that incorporation of P into Pt facilitates CO desorption, as proven by the negative shift of CO oxidation peak (0.11 V) in the CO stripping test (Fig. 4k).

### Scalable neutral H_2_O_2_ electrosynthesis

**Fig. 5:**
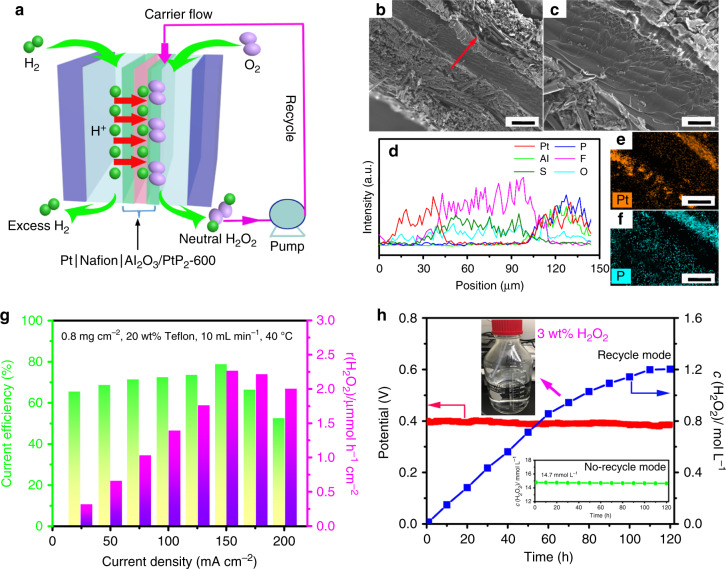
Performance of polymer electrolyte membrane fuel cell (PEMFC). **a** Schematic diagram of PEMFC for O_2_-to-H_2_O_2_ production with product recycling. **b**, **c** Cross-sectional SEM images; scale bar, **b** 100 µm; **c** 20 µm. **d** line-scan elemental distribution, and **e**, **f** elemental mapping of Al_2_O_3_/PtP_2_-600 based MEA; scale bar, 50 µm. **g** Current efficiency and H_2_O_2_ production rate as a function of current density under optimized conditions. **h** Time-dependent neutral H_2_O_2_ concentration measured at a constant potential of 0.4 for 120 h. The accumulated H_2_O_2_ concentration in 600 mL when the product is continuously cycled through the system. Concentration reaches a metric value of 3.0 wt% after 65 h (inset of Fig. 5h).

Having developed a stable and selective electrocatalyst for O_2_-to-H_2_O_2_ conversion, we further incorporated our catalyst into a practical polymer electrolyte membrane fuel cell (PEMFC) to synthesize a neutral H_2_O_2_ solution at high concentration and volume. Figure 5a shows a schematic diagram of a PEMFC using commercial Pt/C anode for H_2_ oxidation, Nafion 117 membrane for proton transportation and elimination of gas crossover, and our Al_2_O_3_/PtP_2_-600 cathode for two-electron ORR. The details of the expeirmental setup and operation are shown in Supplementary Fig. 31. The Al_2_O_3_/PtP_2_-600 catalyst ink was spray coated onto the carbon gas-diffuse layer (GDL) and hot pressed with the Nafion membrane to form the MEA. The overall MEA is compactly connected and no voids are observed between the interfaces (Fig. 5b, c). The Pt, P, and Al elements are well distributed across the MEA (Fig. 5d–f). The optimized conditions (e.g., mass loading, catalyst support, water flow rate, and operation temperature) for H_2_O_2_ production in PEMFC are summarized in Supplementary Table 6. The optimized mass loading of Al_2_O_3_/PtP_2_-600 is determined to be 0.8 mg cm^−2^ (Supplementary Fig. 32), as lower loading leads to kinetics loss and higher loading causes high O_2_ gas mass transport reisistance^41^. The current efficiency (CE%) and H_2_O_2_ production rate (r(H_2_O_2_)) at 50 mA cm^−2^ is 60.8% and 0.57 mmol h^−1^ cm^−2^, respectively. A high CE is achieved despite the more challenging kinetics for H_2_O_2_ production at neutral conditions compared to the commonly used acidic conditions. At high current density, cathode flooding is typically observed in carbon-based GDE due to the loss of hydrophobicity during PEMFC operation^42^. For the PEMFC with 20 wt% teflon-treated GDL, the operating current density can be increased up to 125 mA cm^−2^ with significantly improved r(H_2_O_2_) (1.51 mmol h^−1^ cm^−2^) compared to that with the non-teflon-treated GDE (Supplementary Fig. 33). Both the highest CE% and r(H_2_O_2_) are achieved at a water flow rate of 10 mL min^−1^ (Supplementary Fig. 34). Increasing the water flow rate within 2–10 mL min^−1^ is beneficial to remove the generated H_2_O_2_ and minimize its thermochemical decomposition and/or further electroreduction^27^. However, further increasing the water flow rate to 20 mL min^−1^ reduces the CE% and r(H_2_O_2_), which is ascribed to the O_2_ mass transport being poor under high volume water flow. The optimized temperature is only 40 °C. Increasing operating temperature would improve proton conducticity in the MEA, but the dominant effect is to increase H_2_O_2_ thermochemical decomposition^25^. Overall, the optimized operation conditions of PEMFC for O_2_-to-H_2_O_2_ are catalyst loading of 0.8 mg cm^−2^, 20 wt% teflon-treated carbon GDL, water carrier flow rate of 10 mL min^−1^, and operation temperature of 40 °C. A maximum r(H_2_O_2_) of 2.26 mmol h^−1^ cm^−2^ is obtained with the PEMFC operating at 150 mA cm^−2^ with a highest CE of 78.8% (Fig. 5g). The difference in CE for of O_2_-to-H_2_O_2_ in RRDE and MEA system is attributed by the transport efficiency of H_2_O_2_ away from the electrode surface^2^. In the RRDE, the small amount of generated H_2_O_2_ molecules are rapidly transported away from the disk electrode and oxidized at the ring electrode, leading to a low steady-state surface H_2_O_2_ concentration. By contrast, the H_2_O_2_ molecules produced at the catalyst/membrane interface in the MEA system need a longer time to diffuse though the catalyst layer and gas diffusion layer before removal into the water stream, which causes a higher local concentration of H_2_O_2_ at the vicinity of catalyst surface and increases the possibility of further H_2_O_2_ reduction^27^. Moreover, nearly no degradation of the H_2_O_2_ concentration (14.7 mmol L^−1^, pH = 6.8) is observed at a constant potential of 0.4 V in 120 h (Fig. 5h). The compact interfaces for both cathode/electrolyte and anode/electrolyte are well maintained after 120 h of measurement (Supplementary Fig. 35). For comparison, a Pt–Hg NC-based MEA was fabricated in the same way and run in the PEMFC under the same conditions. The concentration of leached Hg is 5.9 × 10^3^ ppb in neutral H_2_O_2_ catholyte solution (Supplementary Table 7), and approximately 38% degradation for H_2_O_2_ concentration was observed after 6 h of PEMFC operation without recycling of the product (Supplementary Fig. 36). To obtain a neutral H_2_O_2_ solution with high concentration, the product was recycled to run through the catalyst multiple times, following an initial 1 h run to accumulate 600 mL H_2_O_2_ solution. Under recycling the accumulated H_2_O_2_ concentration is increased with the operation time and approaches to 1.21 mol L^−1^ for 120 h. It should be noted that a H_2_O_2_ concentration of 3.0 wt% (pH = 6.6) in 600 mL is achieved after 65 h, sufficient for medical sterilization, chemical synthesis, and food processing. The saturation in the accumulated H_2_O_2_ concentration is attributed to competing thermochemical and/or electrochemical degradation pathways of H_2_O_2_. To elucidate the degradation pathways, the electrolysis under recycled operation was conducted in an O_2_-free 1 M H_2_O_2_ solution at the same conditions in PEMFC. The time-dependent H_2_O_2_ concentration is shown in Supplementary Fig. 37. At open-circuit voltage, the decrease of H_2_O_2_ concentration is mainly caused by thermochemical reduction and only 48% H_2_O_2_ remains after 60 h recycling. At 0.4 V vs. RHE, both the thermochemical and electrochemical reduction processes contribute to H_2_O_2_ degradation. Notably, the electrochemical reduction rate of H_2_O_2_-to-H_2_O is much slower than that of thermochemical reduction, which significantly reduces the consumption of produced H_2_O_2_ and leads to high saturation levels of H_2_O_2_ accumulation during electrolyte recycling. A similar recycling strategy for making high concentration H_2_O_2_ solution from O_2_ electroreduction in PEMFC was also demonstrated in previous work^27^.

In summary, monodisperse colloidal platinum diphosphide (PtP_2_) NCs applied as efficient two-electron ORR electrocatalysts. The PtP_2_ NCs require a near-zero overpotential for H_2_O_2_ initialization and achieve a maximum O_2_-to-H_2_O_2_ selectivity of 98.5% at 0.27 V vs. RHE. DFT results suggest that weakened binding of the OOH* intermediate and inhibition of subsequent O–O breakage of OOH* compared to pure Pt. A PEMFC with Al_2_O_3_/PtP_2_-600 as the cathode catalyst achieves a maximum r(H_2_O_2_) of 2.26 mmol h^−1^ cm^−2^, a CE up to 78.8%, and sufficient stability to achieve an accumulated neutral H_2_O_2_ concentration of 1.21 mol L^−1^ by 120 h. This work provides insight into the development of efficient and stable electrocatalysts for selective production of neutral H_2_O_2_ in a practical device.

## Methods

### Chemicals and materials

Oleylamine (OAm, tech. 70%), oleic acid (OA, tech. 90%), 1-octadecene (ODE, tech. 90%), Nafion 117 solution, Teflon dispersion, and commercial Pt/C (20 wt%) were purchased from Sigma-Aldrich. Platinum(II) 2,4-pentanedionate was from Alfa Aesar. Tris(trimethylsilyl)phosphine ((Me_3_Si)_3_P) was purchased from Strem Chemicals. Commercial carbon black, GDL, and Nafion 117 membrane were obtained from FuelCellStore.

### Synthesis of platinum diphosphide (PtP_2_) NCs

A solution of platinum(II) 2,4-pentanedionate (0.3 mmol), OAm (8 mL), OA (0.5 mL), and ODE (8 mL) was placed into a round bottom flask with a stir bar and degassed at 120 °C under vacuum for 1 h. To prepare for injection of the P source, the solution was then heated to 220 °C under nitrogen. Meanwhile, the P precursor was prepared by placing 1.2 mL (Me_3_Si)_3_P dissolved in hexane (10 wt%) and 1.0 mL ODE under vacuum to remove the hexane at room temperature. The (Me_3_Si)_3_P solution was quickly injected at 220 °C and for 15 min the temperature was maintained. To facilitate Ostwald ripening, during cooling the system was kept at 120 °C for 10 min prior to cooling to room temperature. To purify, the precipitate was collected following centrifugation at 6000 rpm for 6 min and redispersed with hexane. To further purify the NCs, aggregation was induced with a 1:6:1 (v:v:v) hexanes:acetone:methanol solution followed by centrifuged and resuspension with hexane and this procedure was then repeated for a total of four times. The final suspension yield a solution with NC density by weight of 2 mg/mL. For comparison, Pt NCs were synthesized in a similar co-heating way without (Me_3_Si)_3_P injection. For the synthesis of colloidal Pt–Hg NCs, platinum(II) 2,4-pentanedionate (0.2 mmol), and mercury (II) chloride (0.2 mmol), OAm (4 mL), and ODE (4 mL) were added into a 25 mL reaction flask. The solution was degassed at 120 °C for 1 h and then heated to 200 °C where it was held for 2 h. To facilitate Ostwald ripening, during cooling the system was kept at 100 °C for 10 min prior to cooling to room temperature.

### Materials characterizations

Elemental analysis was performed using ICP–MS (Teledyne Leeman Labs). A Bruker D2 Phaser was used to collecte X-ray diffraction (XRD) using Cu Kα radiation. High-resolution transmission electron microscopy (HRTEM) was performed with a Tecnai G2 F20 microscope at 200 or 300 kV. STEM was done using a JEOL JEM 2200FS STEM/TEM microscope at an acceleration voltage of 200 kV equipped with a CEOS probe corrector (Heidelberg, Germany). For EDS a Bruker-AXS silicon drift detector was used. An Autosorb-iQ from Quantachrome was used to find the BET surface area from nitrogen adsorption isotherms. XPS was performed with a Kratos Axis Ultra DLD, UK, and the C 1s peak at 284.8 eV was used for calibration. In situ and ex situ Pt L_3_-edge XAFS measurements were made at the beamline 14W1 in Shanghai Synchrotron Radiation Facility (SSRF), China. PtO_2_, PtCl_2_, and Pt foil were used for linear combination fitting to calculate the Pt valence oxidation state. XANES calculations were done using the FEFF8.2 code and the multiple-scattering method. The experimental absorption coefficients are reported as normalized absorption following background subtraction.

### Catalyst inks and electrode preparation

The NCs were combined with an equal amount by weight of Ketjen carbon (C) and sonicated for 1 h (5 mL of 2 mg mL^−1^ NC solution with 10 mg of C support). To remove excess ligands, the hexane was allowed to evaporate and the catalyst was maintained at 60 °C for 2 h in acetic acid (8 mL). This solution was then centrifuged for 5 min at 5000 rpm following addition of 8 mL of ethanol. The collected precipitate was underwent an additional two cycles of the acetic acid treatment. The final catalyst ink (2 mg mL^−1^) was composed of the purified NCs and isopropanol, water, and Nafion 117 solution (v:v:v = 2:4:0.05). A rotating disk electrode (RDE) with a glassy carbon core was polished by a 0.5 and 0.05 μm alumina powder and rinsed with deionized water. Unless specified, 20 μL of catalyst ink was drop deposited on the glassy carbon working electrode (OD = 6 mm, MTI34 series) with a loading of 0.2 mg cm^−2^.

### Electrochemical measurements

For ORR, all tests were done using a conventional three electrode electrochemical cell with a Biopotentiostat Model AFCBP1 (Pine Instrument Company). The cyclic voltammetry (CV) experiments were performed in N_2_- or O_2_-saturated 0.1 M HClO_4_ at room temperature with a scan rate of 10 mV/s. RDE and rotating ring-disk electrode (RRDE) tests were conducted in O_2_-saturated 0.1 M HClO_4_ with a scan rate of 10 mV/s. The RRDE voltammograms were performed with a glassy carbon disk electrode and a Pt ring electrode. Pt foil and Ag/AgCl were used as counter electrode and reference electrode, respectively. Flow of O_2_ was maintained into the electrolyte during the entire ORR process to ensure the O_2_/H_2_O equilibrium. The disk electrode was scanned at a rate of 10 mV/s at 1600 rpm, and the Pt ring electrode potential was fixed at 1.5 V vs. RHE. The hydrogen peroxide yield (%H_2_O_2_) and electron transfer number (n) were calculated by the following equations:

1 $${\mathrm{\% }}{\mathrm{H}}_{2}{\rm{O}}_{2} = 200\frac{{i_r/N}}{{i_d + i_r/N}} = 200\frac{{i_r}}{{Ni_d + i_r}},$$

2 $${n} = 4\frac{{i_d}}{{i_d + i_r/N}} = 4\frac{{Ni_d}}{{Ni_d + i_r}},$$

where *i*_*d*_ and *i*_*r*_ are the disk and ring currents, respectively. *N* is the Pt ring current collection efficiency. The *N* value in our system was calibrated in 0.1 M HClO_4_ with a 10 mM K_3_Fe(CN)_6_ electrolyte and is approximately 0.25 (Supplementary Fig. 38). For the CO striping test, the electrodes were initially immersed in the CO-saturated 0.1 M HClO_4_ by purging with 10 wt% CO in N_2_ gas for 30 min and then set to 0.10 V vs. RHE for 15 min to form a CO adsorption layer on the catalyst surface. Then the electrolyte was purged by N_2_ gas for 10 min to remove the remaining CO in solution. The CO stripping CVs were obtained in a potential range of 0–1.2 V with a scan rate of 20 mV/s. Electrochemical impedance spectroscopy was performed on a Reference 600 (Gamry Instrument Inc.) with the working electrode biased at OCP, while sweeping the frequency from 10^5^ to 0.1 Hz with a 10 mV AC dither. To determine the electrochemical capacitance, CV scans in the non-faradaic potential region were conducted and the capacitive current was obtained at the middle potential value for each scan rate. CV was carried out in a nitrogen-purged 5 mM K_3_Fe(CN)_6_/0.1 M HClO_4_ solution with platinum foil as the counter electrode. EASA values were calculated using the Randles–Sevcik equation^43^:

3 $$I_p = \left( {2.36 \times 10^5} \right)n^{3/2}AD^{1/2}C\upsilon ^{1/2},$$

where *I*_*p*_ is peak current (*A*), *n* = 1, *D* = 4.34 × 10^−6^ cm^2^ s^−1^, *A* is the EASA (cm^2^), *C* is the concentration of potassium ferricyanide (5 × 10^−6^ mol cm^−2^), and *υ* is the scan rate (5 mV s^−1^). Conversion from vs. Ag/AgCl to vs. RHE was done by adding 0.197 + 0.059 × pH.

For in situ ATR-FTIR measurements, a diamond-like carbon was coated onto a Si wafer (5 × 8 × 1 mm^3^) to prepare the internal reflection element (IRE). The coated IRE was ultrasonicated for 2 min with 30% concentrated H_2_SO_4_ followed by rinsing with DI water before experiments. A 50 µL of 2 mg mL^−1^ catalyst ink (no Nafion binder) was dropcast on the IRE and dried under air at room temperature. A glassy carbon paper was placed on top of the catalyst layer for good electrical contact. Glassy carbon rod connected to the IRE, Pt gauze, and Ag/AgCl in 3 M KCl were used as the working electrode, counter electrode, and reference electrode, respectively. An FTIR spectrometer with a MCT detector was used for the in situ ATR-FTIR measurements. Solutions were saturated either with O_2_ for ORR or with Ar as a control. Gamry Reference 600 potentiostat during recording of the IR spectra.

### ALD Al_2_O_3_ overcoat for stabilization

The ALD Al_2_O_3_ overcoat for supported PtP_2_ was grown in a GEMSTAR-6 atomic layer deposition (ALD) system using trimethylaluminum (TMA) and distilled water (H_2_O) at 175 °C. The precursors were kept in the chamber for 2.2 s and a 28 s purge was used. To ensure the deposition occurred with typical growth per cycle, a silicon wafer with native oxide was included alongside the sample as a control and the Al_2_O_3_ thickness on the wafer was determined by the X-ray reflectivity. After 42 cycles, the overcoated PtP_2_ was activated at 600 °C for 2 h in tube furnace under N_2_ gas flow (Al_2_O_3_/PtP_2_-600). For comparison, the pure Al_2_O_3_ thin film with around 2 nm thickness on silicon was fabricated by ALD for 20 cycles.

### Scalable H_2_O_2_ production in fuel cell

The MEA for testing the activity in a H_2_–O_2_ fuel cell was prepared using Al_2_O_3_/PtP_2_-600 catalyst on GDL as cathode, Pt/C catalyst on GDL as anode, and Nafion 117 membrane. To prepare the cathode, a catalyst ink composed of Al_2_O_3_/PtP_2_-600 dispersed in a water–ethanol mixture with ionomer (Nafion solution, 5 wt%) was sprayed on Teflon-treated or non-Teflon-treated GDL. Anode was prepared with commercial Pt/C (20 wt%) catalyst in the same manner as the cathode. The catalyst loading amount for cathode and anode is 0.8 mg_PtP2_ cm^−2^ and 0.3 mg_Pt_ cm^−2^, respectively. A hot press (120 °C and 40 MPa for duration of 5 min) was used to press the components together with a Nafion 117 membrane. The MEA was then assembled in a single fuel cell consisting 4 cm^2^ serpentine flow fields. Humidification of the MEA was performed for 60 min by flowing N_2_ with 100% relative humidity at a cell temperature of 80 °C. The flow rates for H_2_ and O_2_ gases are 150 and 200 mL min^−1^, respectively. The flow rate of external neutral water was controlled by a peristaltic pump. The pure water flow through the cathode chamber is beneficial for the removal of generated H_2_O_2_ molecules and to decrease the thermochemical decomposition and/or further electroreduction of H_2_O_2_. In the product recycling mode of operation, initially the system is run for 1 h run to accumulate 600 mL H_2_O_2_ solution which is then continuously cycled back through the system without any separation of the H_2_O_2_. All the conditions, such as mass loading, catalyst support, water flow rate, and operation temperature, were optimized for H_2_O_2_ production in the PEMFC. For quantitative analysis of H_2_O_2_ concentration, the interaction of the H_2_O_2_ with a modified iodate solution was monitored with the UV–vis spectrscopy method^44^. Briefly, solution A for the I_3_^−^ method consisted of 33 g of KI, 1 g of NaOH, and 0.1 g of ammonium molybdate tetrahydrate diluted to 500 mL with water. The solution was stirred for ~10 min to dissolve the molybdate. Solution A was kept in the dark to inhibit the oxidation of I^−^. Solution B, an aqueous buffer, contained 10 g of KHP per 500 mL. The pH was measured using a pH meter. Equal weights of A and B was subsequently mixed, followed by addition of the H_2_O_2_ solution. The absorbance of the resulting solution was measured at a maximum wavelength of 351 nm.

Based on the obtained concentration of flow H_2_O_2_ solution, the CE of H_2_O_2_ production can be calculated by the following equation:

4 $${\mathrm{CE\% }} = \frac{{2FQC}}{I} \times 100{\mathrm{\% }},$$

where *F* is Faraday’s constant (96485 C mol^−1^), *Q* is the water flow rate (L s^−1^), *C* is the H_2_O_2_ concentration (mol L^−1^), and *I* is the current (A). The corresponding H_2_O_2_ production rate can be expressed as following equation:

5 $${\mathrm{r}}\left( {\rm{H}}_{2}{\rm{O}}_{2} \right) = 3600QC/A,$$

where *A* is the MEA area (4 cm^2^). Full details of experimental procedures can be found in the Supplementary Information.

## Supplementary information

Supplementary Information

## Data Availability

The data that support the findings of this study are available from the corresponding author upon reasonable request.
