# Supplementary material for: Scalable neutral H2O2 electrosynthesis by platinum diphosphide nanocrystals by regulating oxygen reduction reaction pathways
Source: Nat Commun. 2020 Aug 6;11:3928. doi: 10.1038/s41467-020-17584-9 (PMC7411044; doi:10.1038/s41467-020-17584-9)
Supplement: Supplementary file 1 — Supplementary Information [file 41467_2020_17584_MOESM1_ESM.pdf]

Supplementary information for "Scalable neutral H_2O_2 electrosynthesis by platinum diphosphide nanocrystals by regulating oxygen reduction reaction pathways" by Hui Li et al.

Supplementary Figure 1

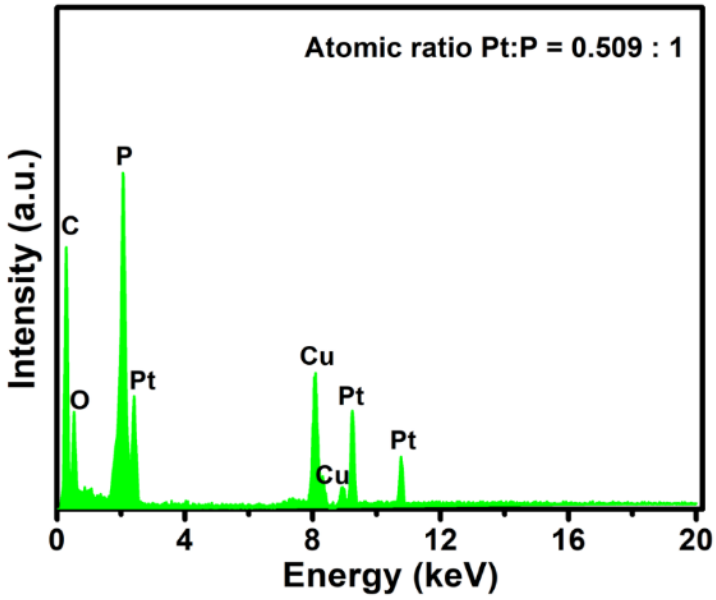

Supplementary Figure 1. Energy-dispersive X-ray spectroscopy (EDS) of PtP_2 NCs. Sample on TEM copper grid.

Supplementary Figure 2

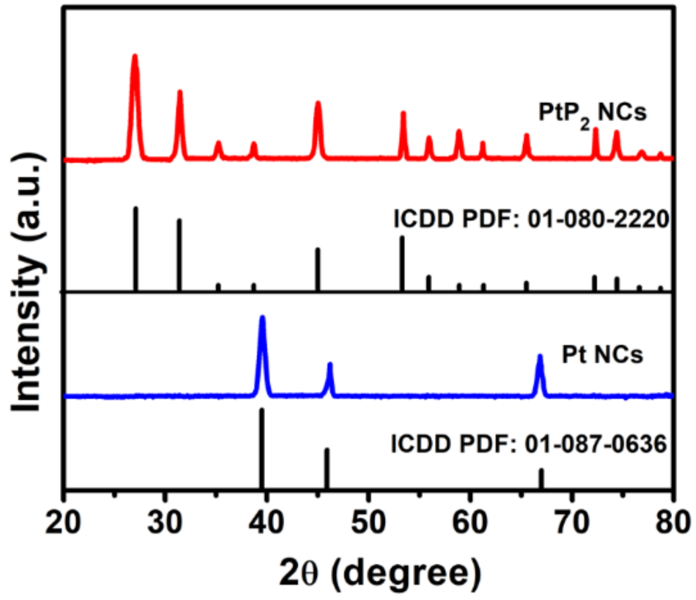

Supplementary Figure 2. X-ray diffraction (XRD) patterns of PtP_2 and Pt NCs.

Supplementary Figure 3

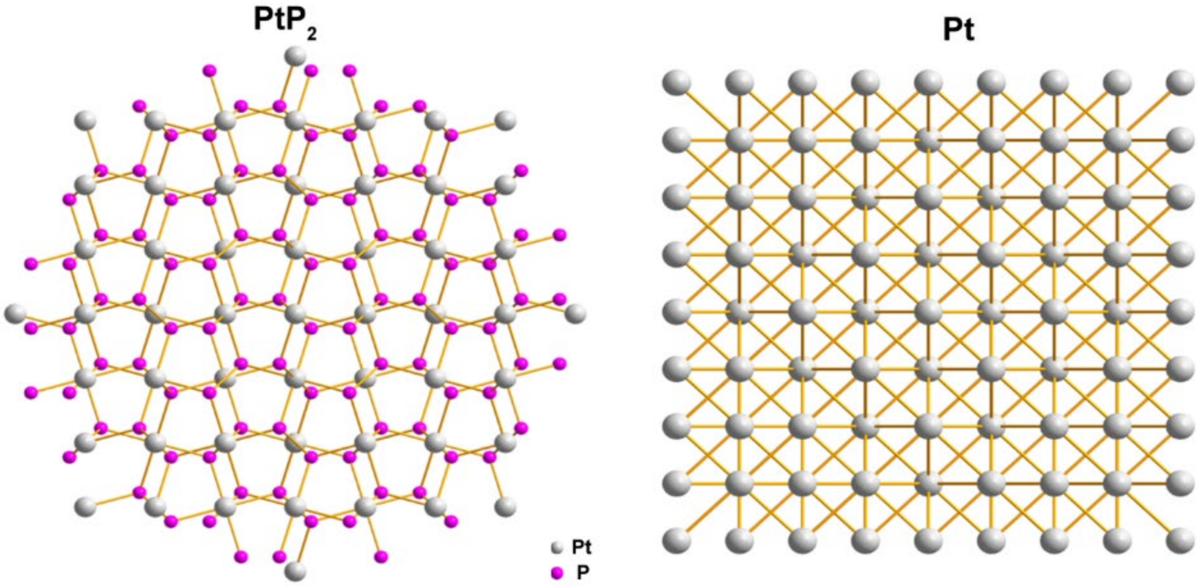

Supplementary Figure 3. Crystal structures of cubic PtP_2 and Pt NCs.

Supplementary Figure 4

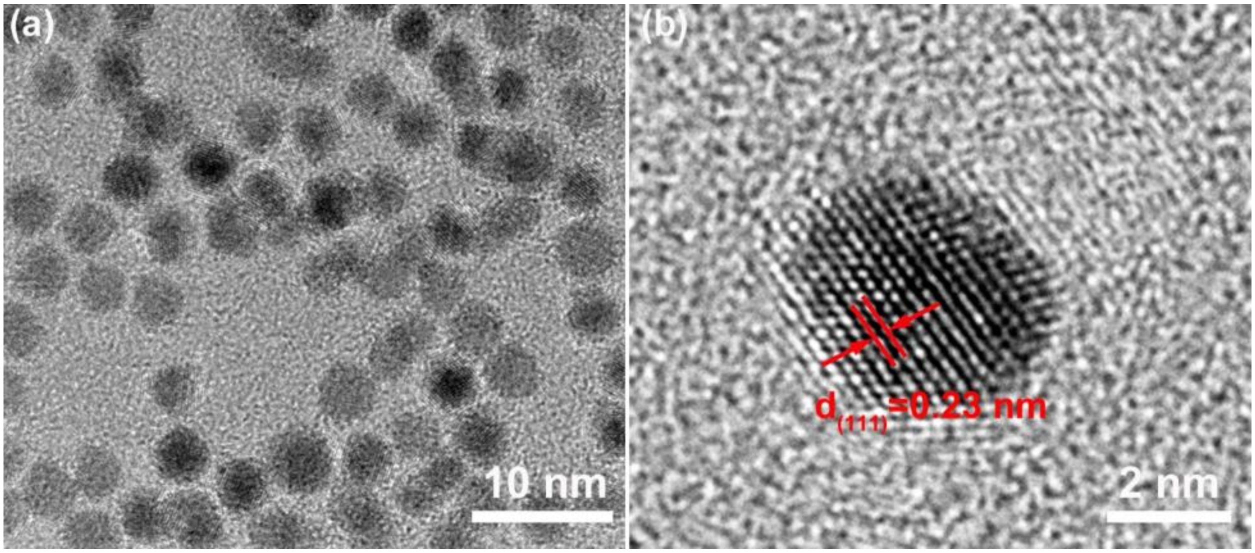

Supplementary Figure 4. Electron microscopy characterization of Pt NCs. **(a)** TEM and (b) HRTEM images of Pt NCs for comparison. The average size of Pt NCs is 4 ± 0.3 nm.

Supplementary Figure 5

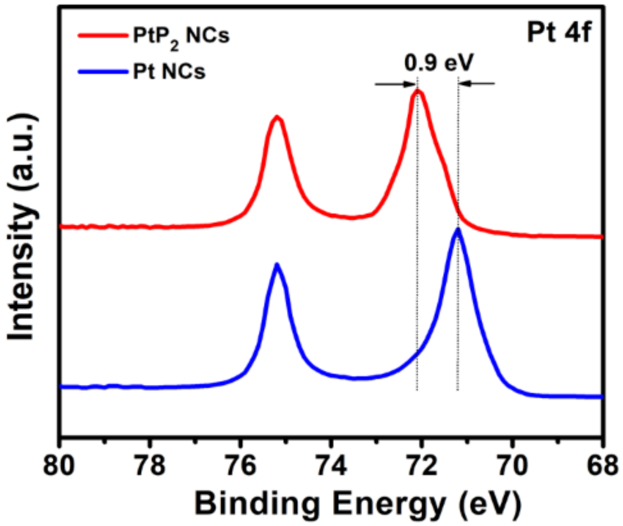

Supplementary Figure 5. Pt 4f core level XPS spectra of PtP_2 and Pt NCs. A positive shift of 0.9 eV for binding energy is observed on PtP_2 NCs compared to the Pt NCs.

Supplementary Figure 6

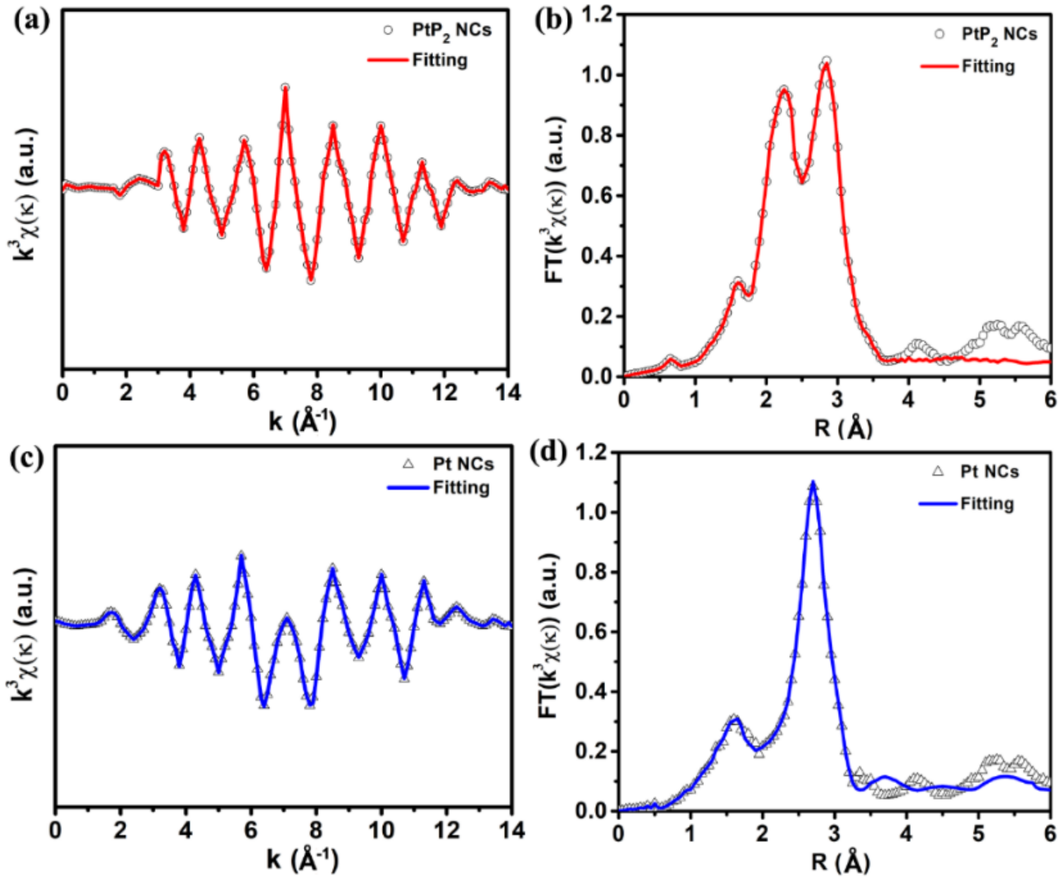

Supplementary Figure 6. R space and inverse FT-EXAFS fitting results of Pt L_3-edge. (a) and (b) PtP_2 NCs, and (c) and (d) Pt NCs. The FT range: 2.0-14.0 $Å^{-1}$; fitting range: 0.5-3.0 Å.

Supplementary Figure 7

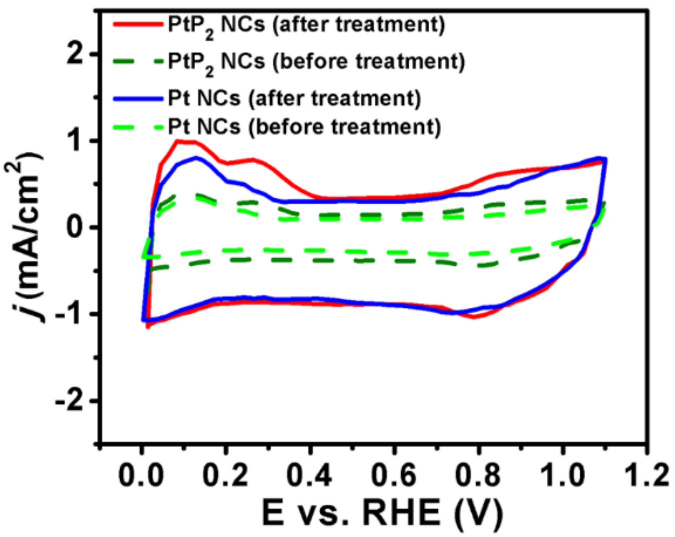

Supplementary Figure 7. Cyclic voltammetry for PtP_2 and Pt NCs before and after acetic acid treatment. Samples in N_2-saturated 0.1 M $HClO_4$. Both the electrochemical activity of PtP_2 NCs and Pt NCs is increased after acetic acid post-treatment. This is consistent with previous work that the mild acetic acid treatment is capable of removing partial surface organic ligand of oleyamine and maintaining the size and surface properties of nanoparticles[1,2].

Supplementary Figure 8

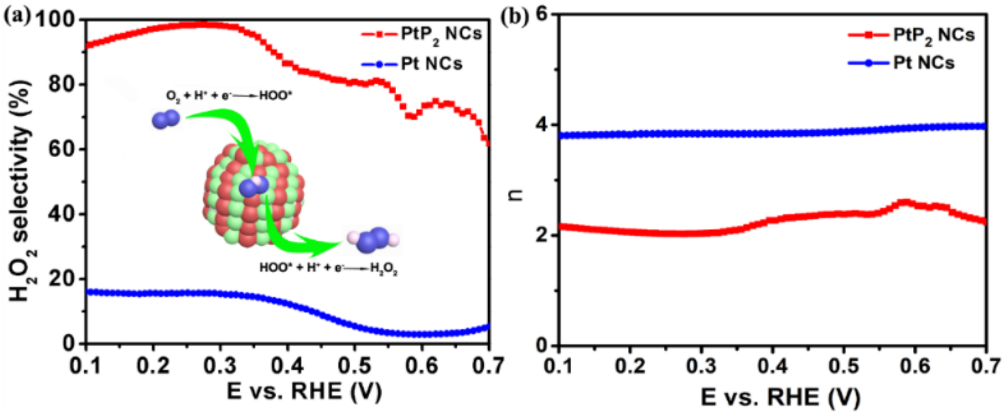

Supplementary Figure 8. Selectivity comparison of PtP_2 and Pt NCs. (a) The percentage of hydrogen peroxide and (b) electron transfer number of PtP_2 and Pt NCs within the scan range of 0.1-0.7 V vs. RHE. The electrolyte is 0.1 M $HClO_4$ (pH = 1).

Supplementary Figure 9

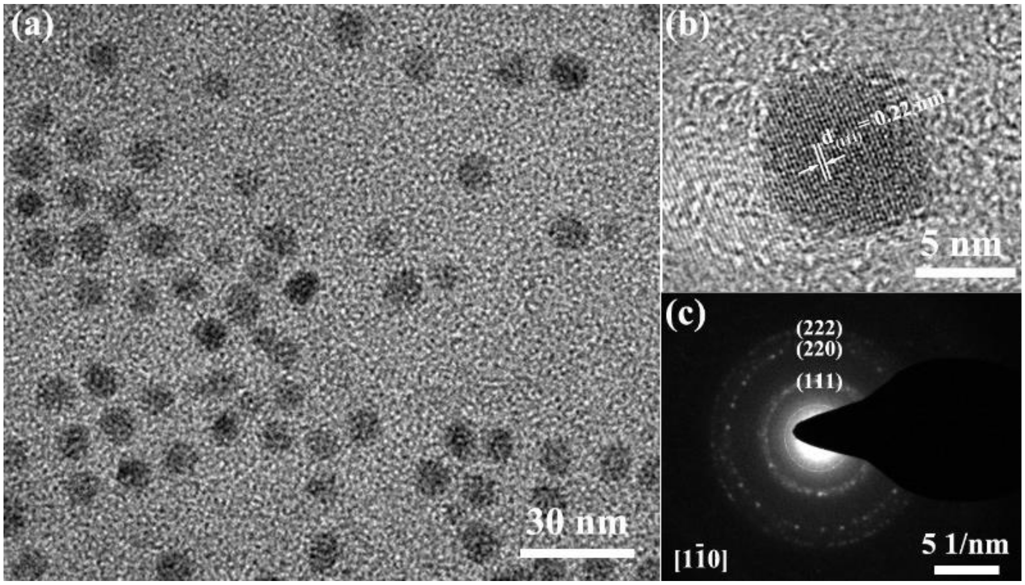

Supplementary Figure 9. Electron microscopy characterization of Pt-Hg NCs. (a) TEM image, (b) HRTEM image, and (c) corresponding selected-area electron diffraction (SAED) image of Pt-Hg NCs.

Supplementary Figure 10

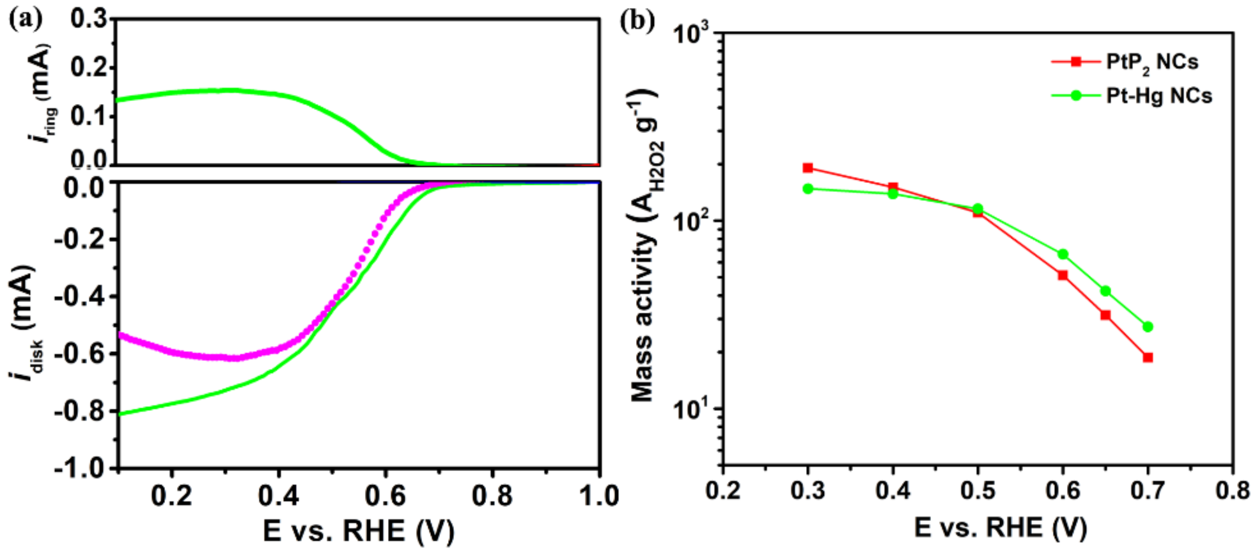

Supplementary Figure 10. Activity measurements of Pt-Hg NCs. (a) RRDE voltammograms of Pt-Hg NCs measured at 1600 rpm in O_2-saturated 0.1 M $HClO_4$ electrolyte. (b) Mass activity of PtP_2 NCs and Pt-Hg NCs for H_2O_2 production in the potential range of 0.3-0.7 V vs. RHE.

Supplementary Figure 11

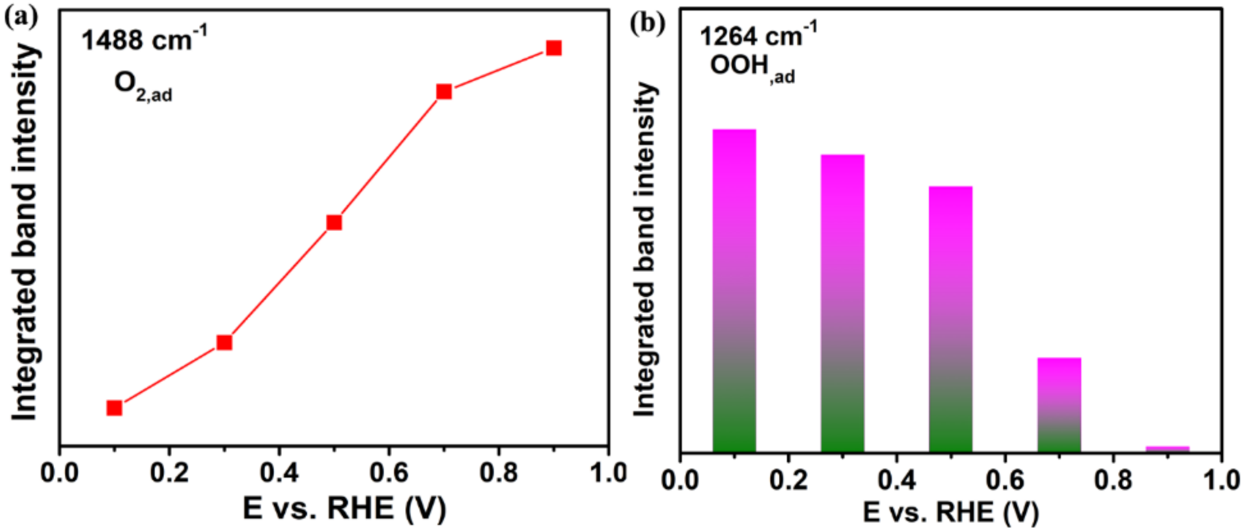

Supplementary Figure 11. In situ ATR- IR during ORR. The integrated intensity of the (a) 1488 cm^{-1} and (b) 1264 cm^{-1} bands at various ORR potentials in O_2- saturated solutions of 0.1 M $HClO_4$ during in situ ATR- IR experiments.

Supplementary Figure 12

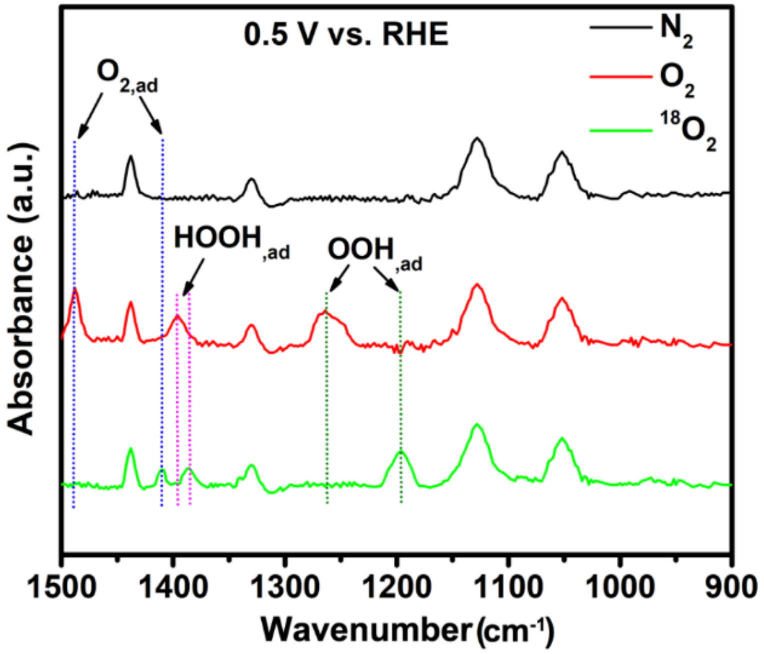

Supplementary Figure 12. IR spectra for supported PtP_2 catalyst in N_2, O_2, and $^{18}O_2$-saturated 0.1 M $HClO_4$ solution at 0.5 V vs. RHE.

Supplementary Figure 13

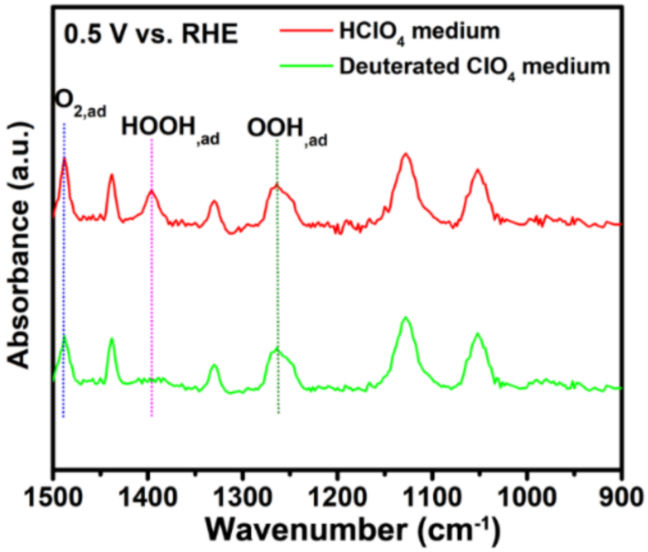

Supplementary Figure 13. The IR spectra for supported PtP_2 catalyst in O_2-saturated 0.1 M $HClO_4$ and deuterated ClO_4^- solution at 0.5 V vs. RHE. Anhydrous $NaClO_4$ was used in D_2O solution saturated with O_2 gas at pD = 1

Supplementary Figure 14

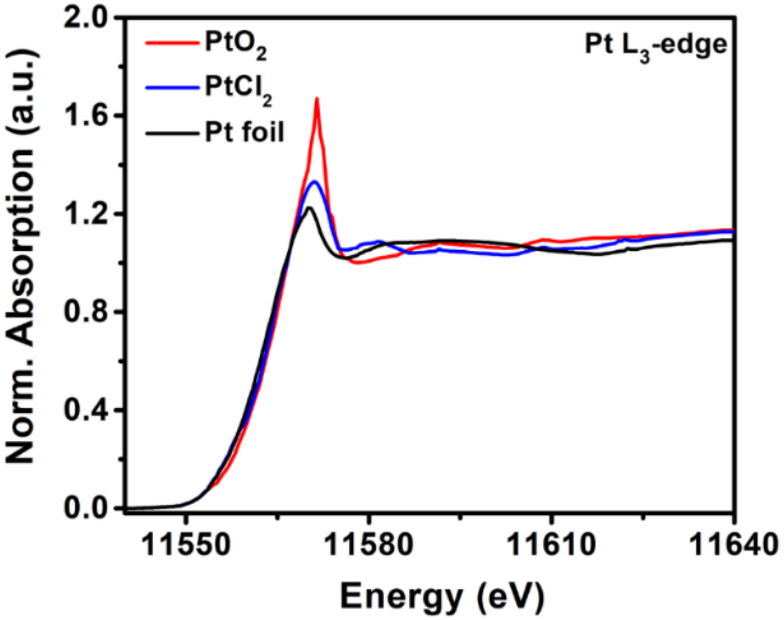

Supplementary Figure 14. Pt L_3-edge XANES spectra of reference samples of PtO_2, $PtCl_2$, and Pt foil.

Supplementary Figure 15

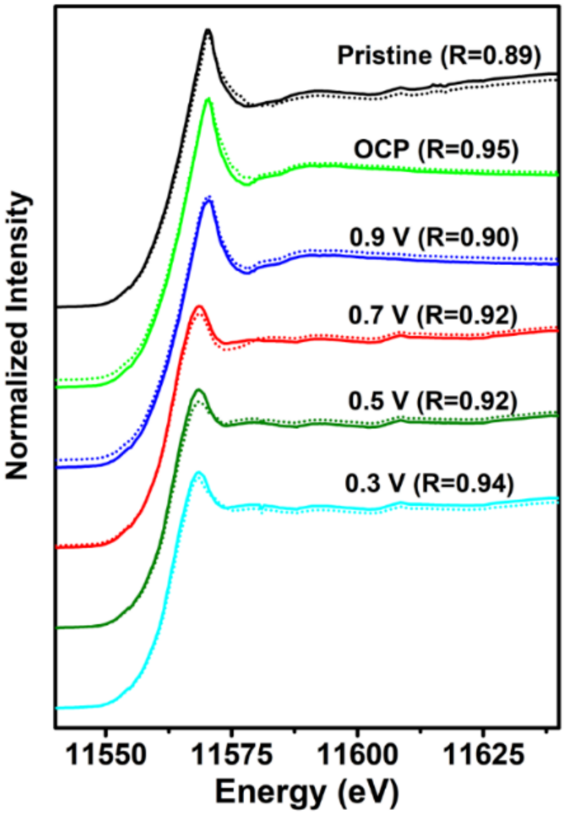

Supplementary Figure 15. In-situ Pt L_3-edge of PtP_2 NCs. Linear combination of PtO_2, $PtCl_2$, and Pt foil spectra (solid line) as compared to the raw in-situ Pt L_3-edge of PtP_2 NCs at OCP (green), 0.9 V (blue), 0.7 V (red), 0.5 V (olive), and 0.3 V (cyan) for 30 min.

Supplementary Figure 16

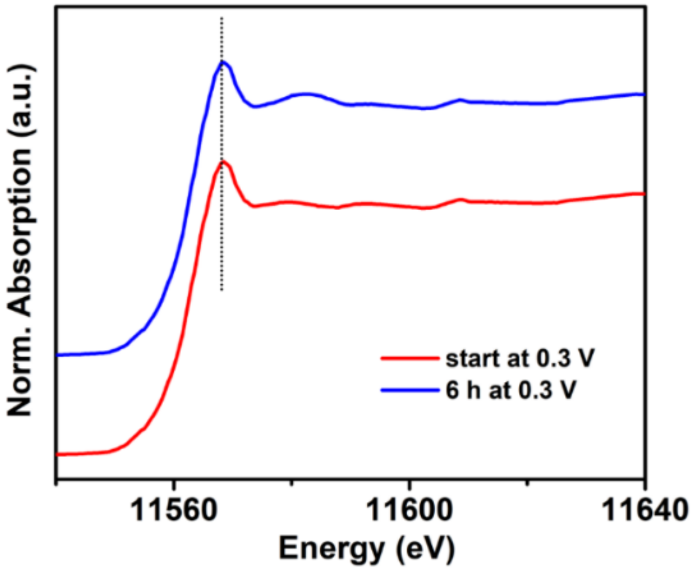

Supplementary Figure 16. Pt L_3-edge XANES spectra of PtP_2 NCs under a constant applied potential of 0.3 V vs. RHE. Initial spectrum and spectrum after 6 h are shown.

Supplementary Figure 17

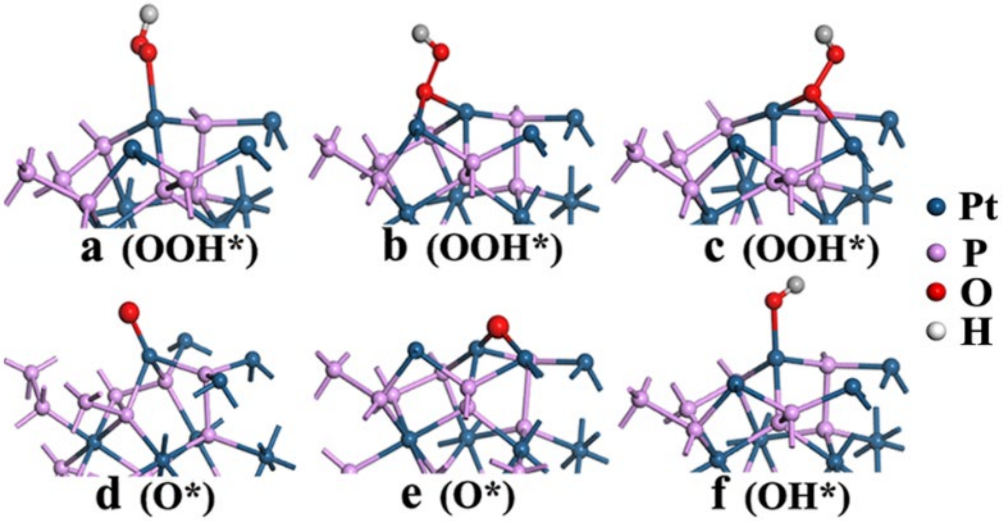

Supplementary Figure 17. Adsorption structures for all possible active sites on PtP_2 (111).

Supplementary Figure 18

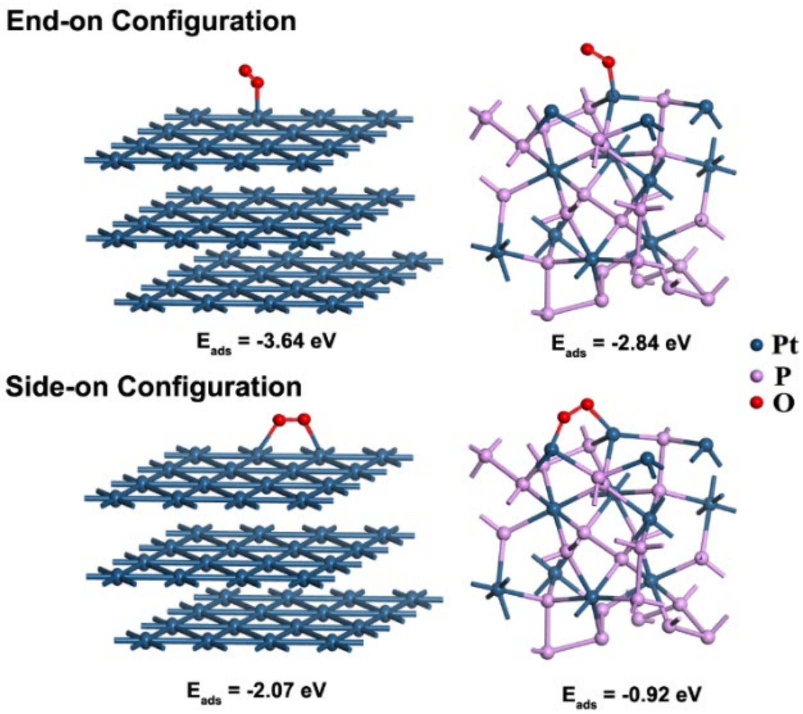

Supplementary Figure 18. O_2 adsorbed on PtP_2 (111) and Pt (111) in the end-on and side-on configuration.

Supplementary Figure 19

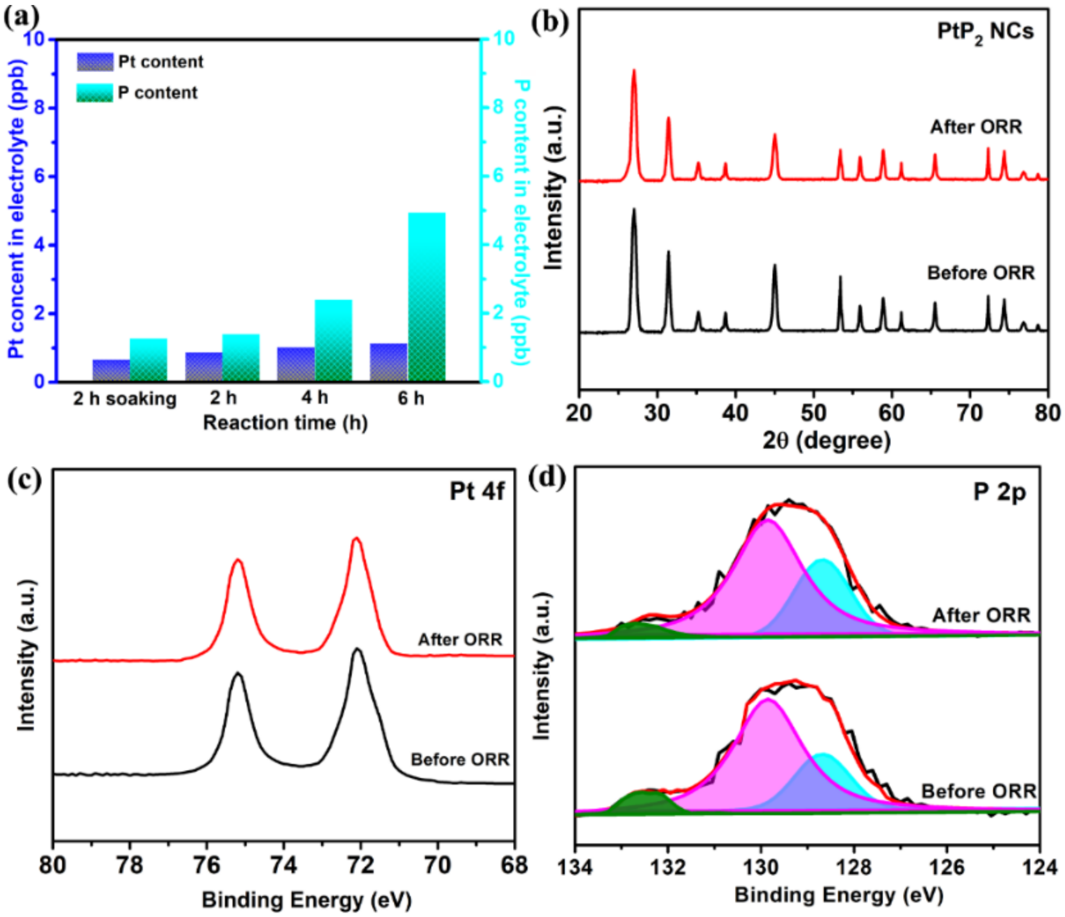

Supplementary Figure 19. Effect of extended ORR on structure. (a) Pt and P content in electrolyte, (b) XRD patterns, (c) Pt 4f, and (d) P 2p core levels of PtP_2 NCs before and after ORR under 0.4 V vs. RHE for 60 h in 0.1 M $HClO_4$.

Supplementary Figure 20

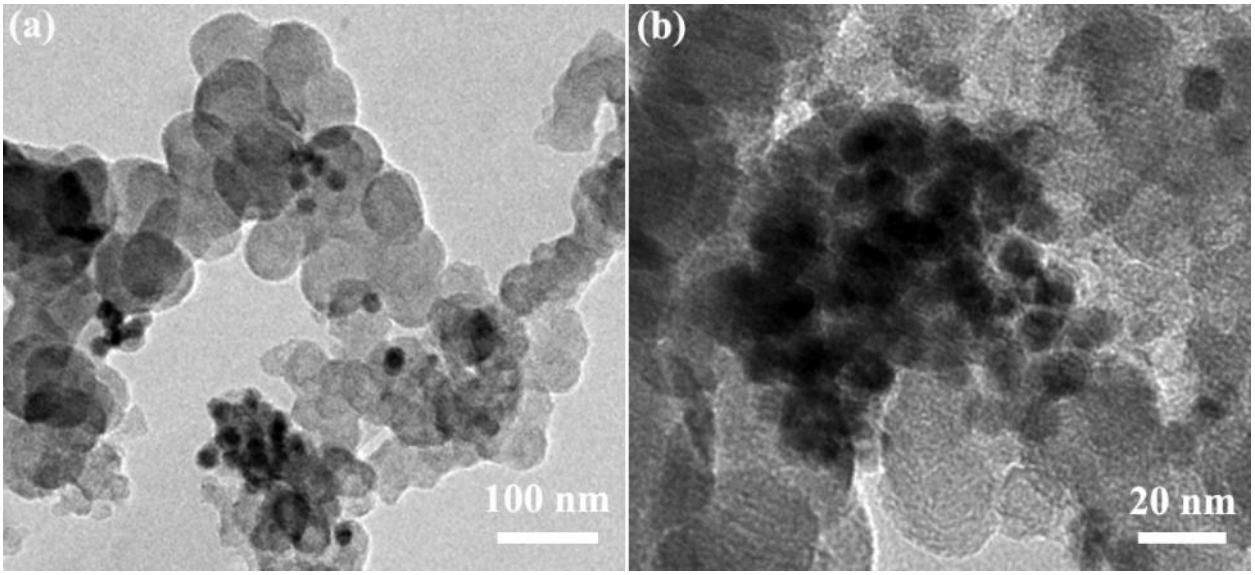

Supplementary Figure 20. Effect of extended ORR characterized by electron microscopy. (a) Low magnitude and (b) high magnitude TEM images of supported PtP_2 NCs after ORR for 60 h at 0.4 V vs. RHE in 0.1 M $HClO_4$.

Supplementary Figure 21

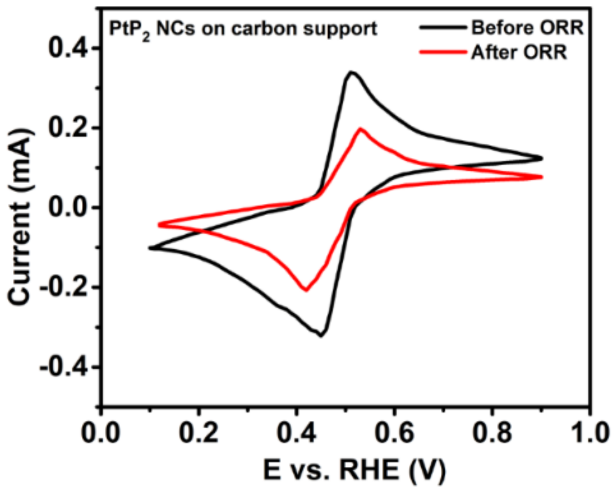

Supplementary Figure 21. Cyclic voltammetry curves of electrochemical active surface area (EASA) of supported fresh-PtP_2 and post-ORR PtP_2 in 5 mM $K_3Fe(CN)_6$/0.1 M $HClO_4$ solution with scan rate of 5 mV s^{-1}.

Supplementary Figure 22

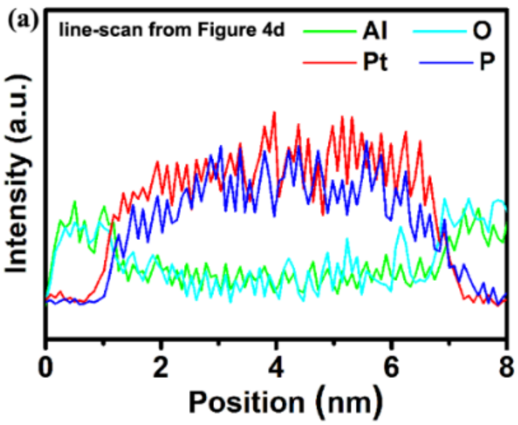

Supplementary Figure 22. High resolution characterization of elemental distribution in NC. Line-scan EDS from (a) Figure 4d and (b) Figure 4f. Carbon signal was removed for better comparison.

Supplementary Figure 23

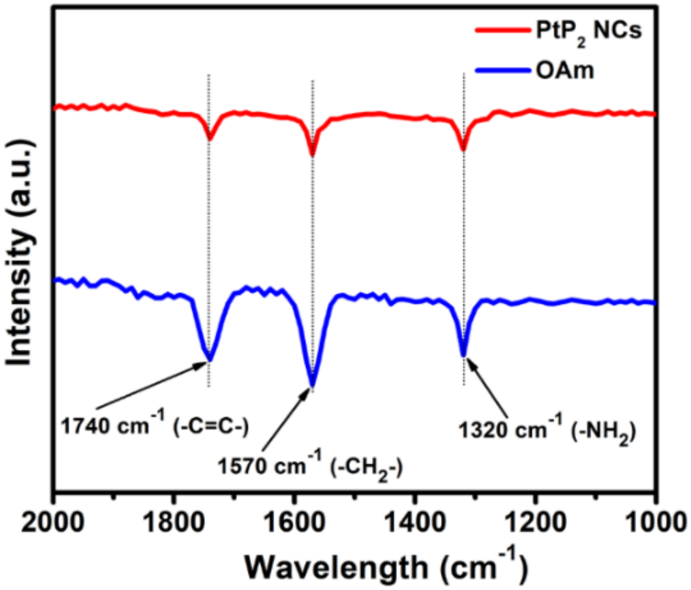

Supplementary Figure 23. FTIR spectra of pure PtP_2 NCs and organic ligand oleyamine (OAm).

Supplementary Figure 24

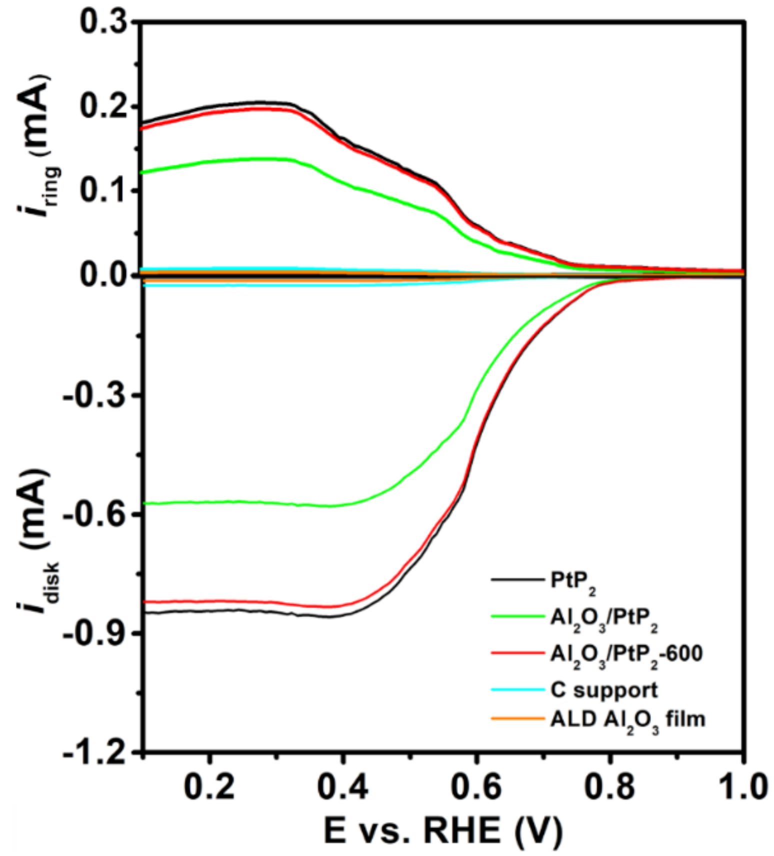

Supplementary Figure 24. Effect of ALD coating on ORR performance. RRDE voltammograms at 1600 rpm in O_2-saturated electrolyte with the disc current and ring current for PtP_2, Al_2O_3/PtP_2, Al_2O_3/PtP_2-600, and control samples of C support and ALD Al_2O_3 thin film.

Supplementary Figure 25

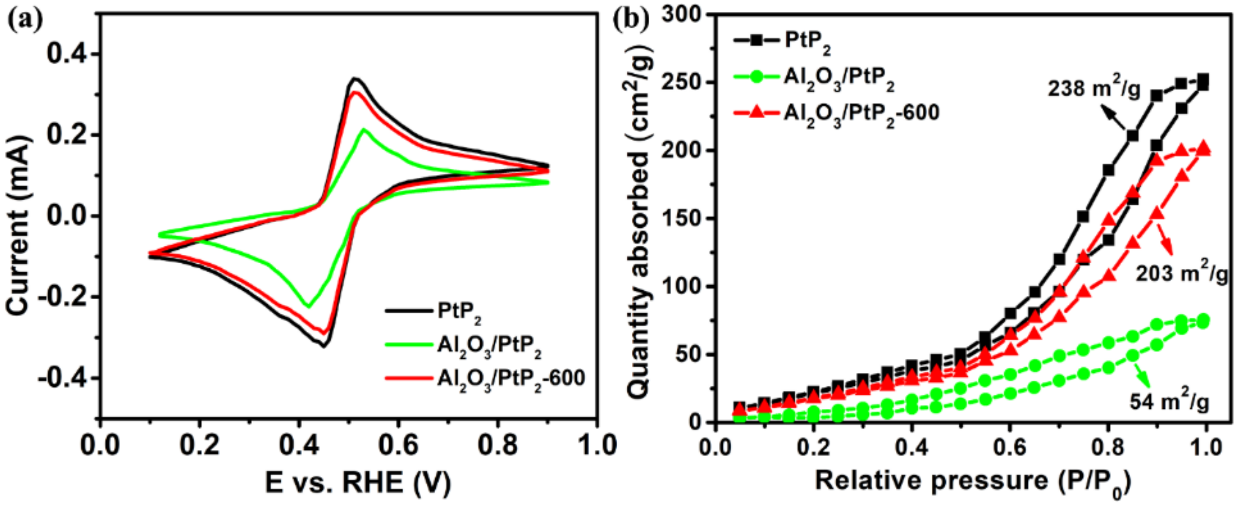

Supplementary Figure 25. Effect of ALD coating characterized by cyclic voltammetry and BET surface area. (a) Cyclic voltammetry curves of electrochemical active surface area (EASA) of supported PtP_2, Al_2O_3/PtP_2, and Al_2O_3/PtP_2-600 in 5 mM $K_3Fe(CN)_6$/0.1 M $HClO_4$ solution with scan rate of 5 mV s^{-1} and (b) BET surface area for PtP_2, Al_2O_3/PtP_2, and Al_2O_3/PtP_2-600.

Supplementary Figure 26

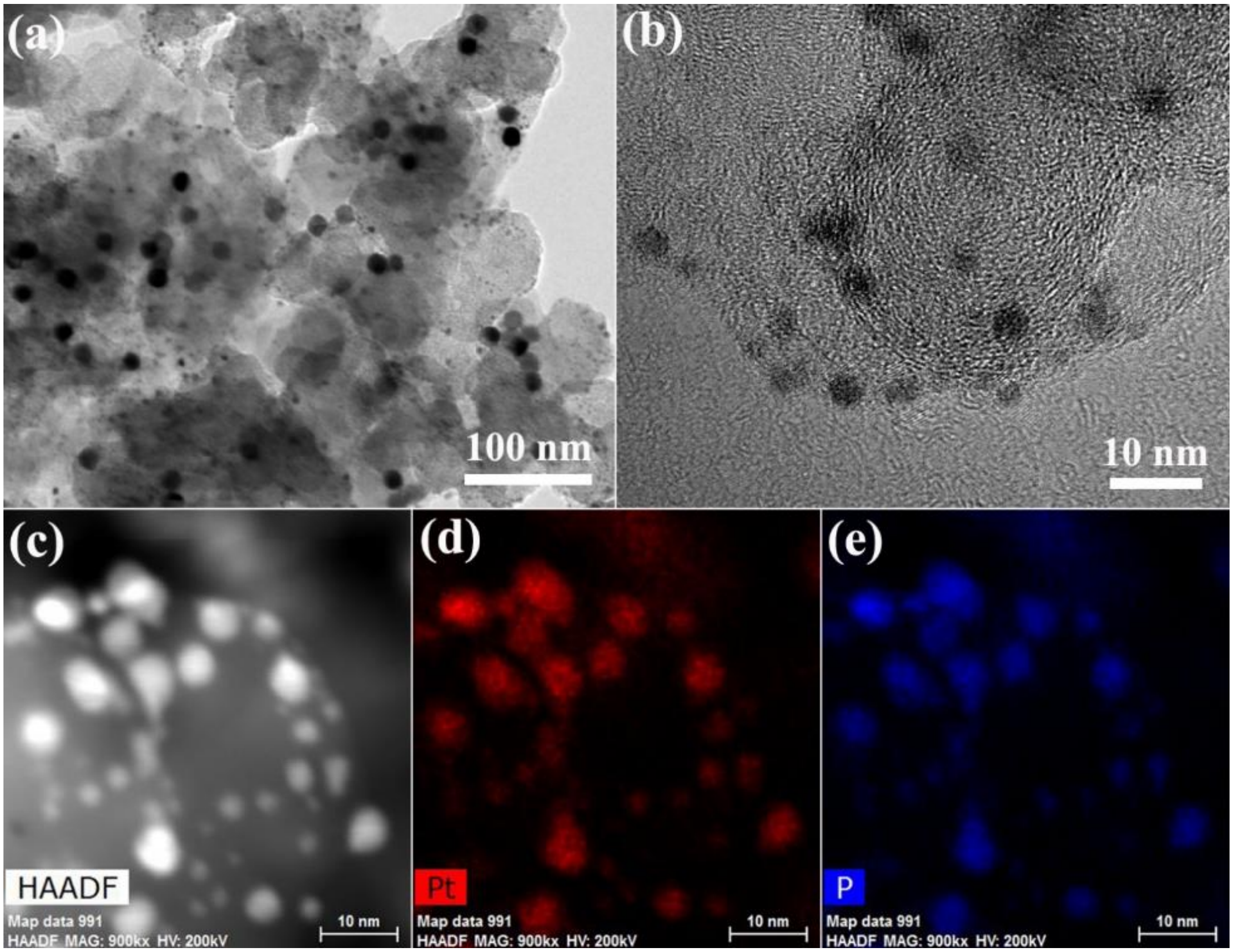

Supplementary Figure 26. Electron microscopy characterization of ALD coated NCs. (a) TEM, (b) HRTEM, (c) HAADF-STEM, and (d,e) elemental mapping for supported Al_2O_3/PtP_2-600 electrocatalyst after ORR measurement at 0.4 V vs. RHE for 60 h.

Supplementary Figure 27

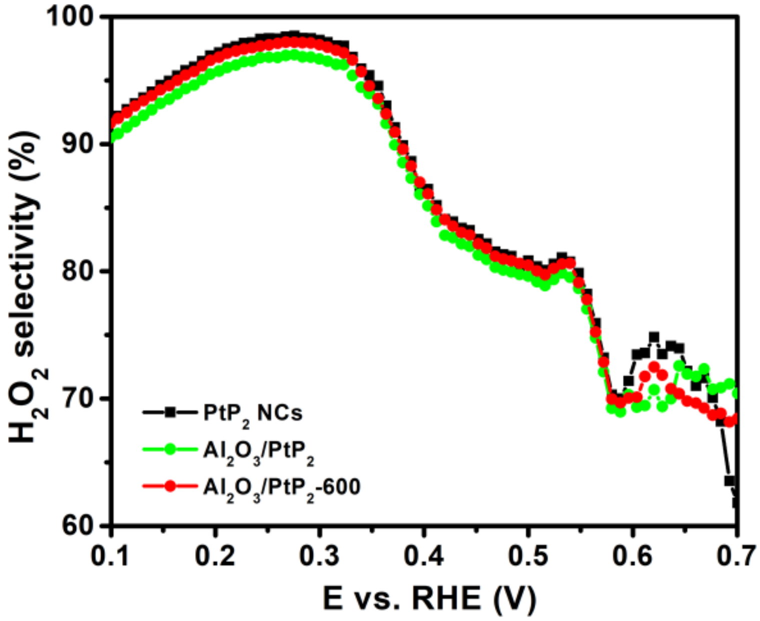

Supplementary Figure 27. The percentage of hydrogen peroxide of PtP_2, Al_2O_3/PtP_2, and Al_2O_3/PtP_2-600 within the scan range of 0.1-0.7 V vs. RHE. The electrolyte is 0.1 M $HClO_4$ (pH = 1).

Supplementary Figure 28

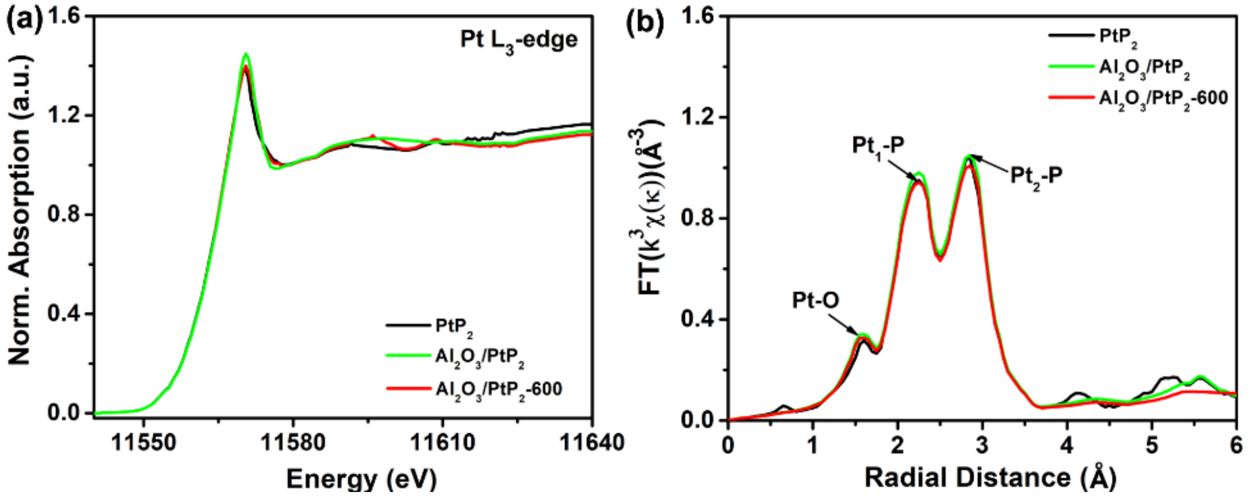

Supplementary Figure 28. XANES and EXAFS characterization of pre- and post-ALD samples. (a) Pt L_3-edge XANES and (b) EXAFS spectra of as-prepared PtP_2, Al_2O_3/PtP_2, and Al_2O_3/PtP_2-600.

Supplementary Figure 29

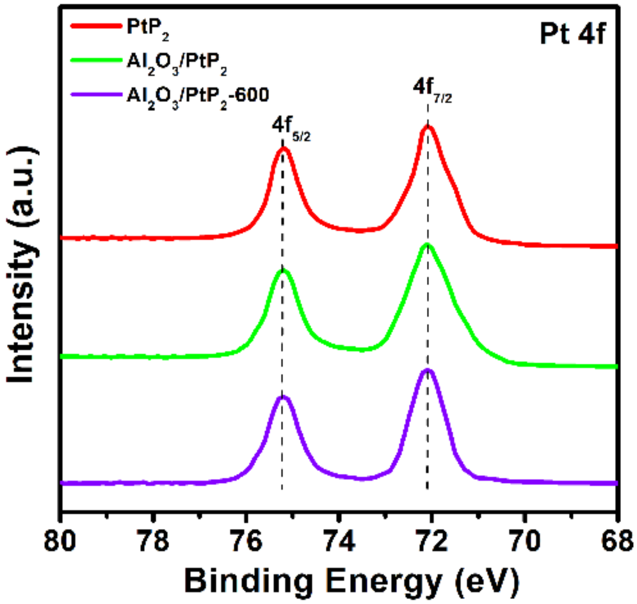

Supplementary Figure 29. Pt 4f core level of PtP_2, Al_2O_3/PtP_2, and Al_2O_3/PtP_2-600.

Supplementary Figure 30

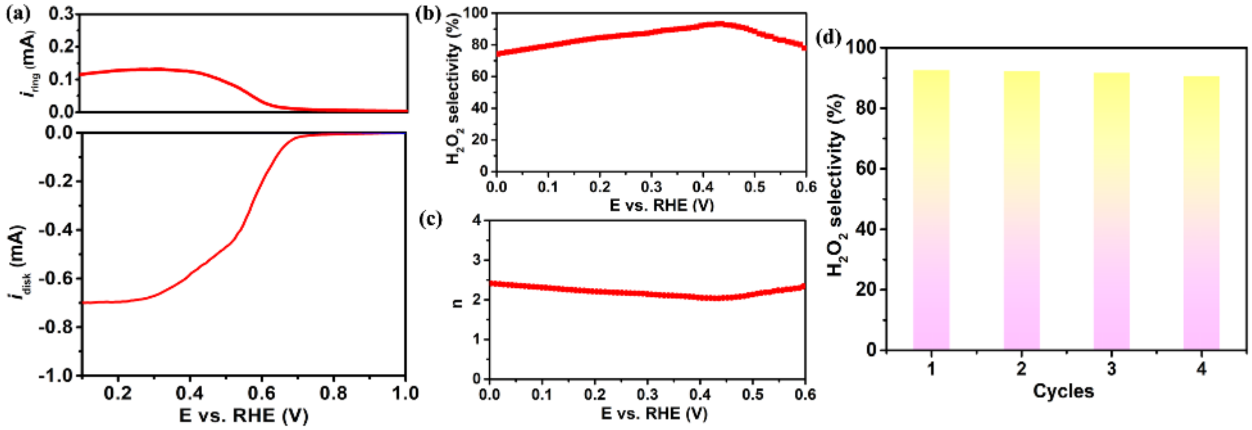

Supplementary Figure 30. RRDE voltammograms of Al_2O_3/PtP_2-600 measured at 1600 rpm in O_2-saturated 0.5 K_2SO_4 electrolyte (pH =7). (a) Linear sweep voltammetry with disk and ring current. (b) H_2O_2 selectivity and (c) electron transfer number as a functional of applied potential. (d) Cyclic stability.

Supplementary Figure 31

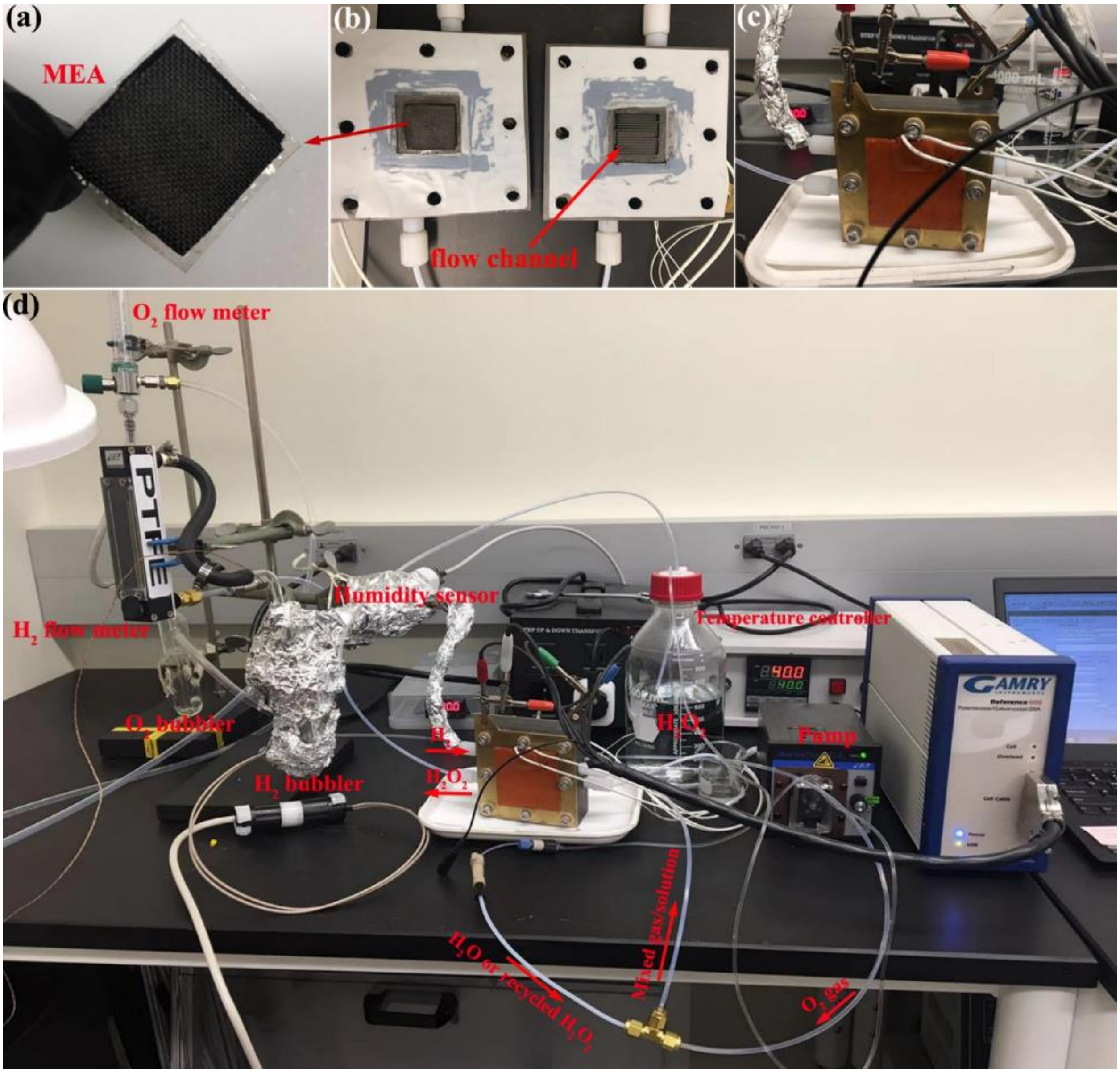

Supplementary Figure 31. Diagram of experimental apparatus. (a) Membrane electrode assembly (MEA), (b,c) single fuel cell reactor, and (d) overall H_2-O_2 fuel cell set-up for direct electrosynthesis of neutral H_2O_2.

Supplementary Figure 32

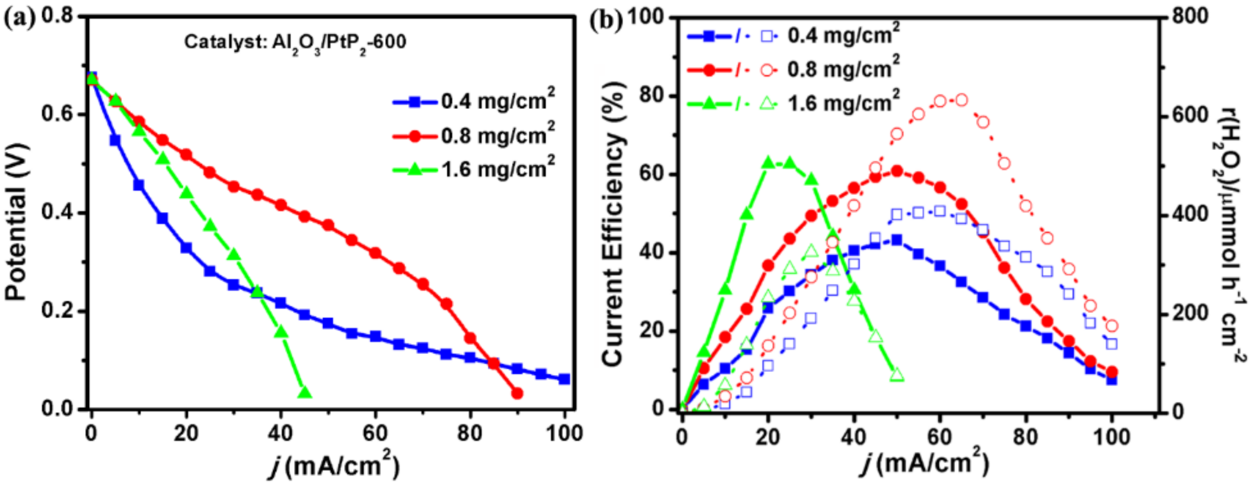

Supplementary Figure 32. Effect of catalyst loading. (a) *j*-*V* polarization curves and (b) current efficiency and H_2O_2 production rate as a function of current density under various cathode catalyst loading. Cathodic conditions: no Teflon, water flow rate of 5 mL min^{-1}, and fuel cell operation temperature of 80 °C.

Supplementary Figure 33

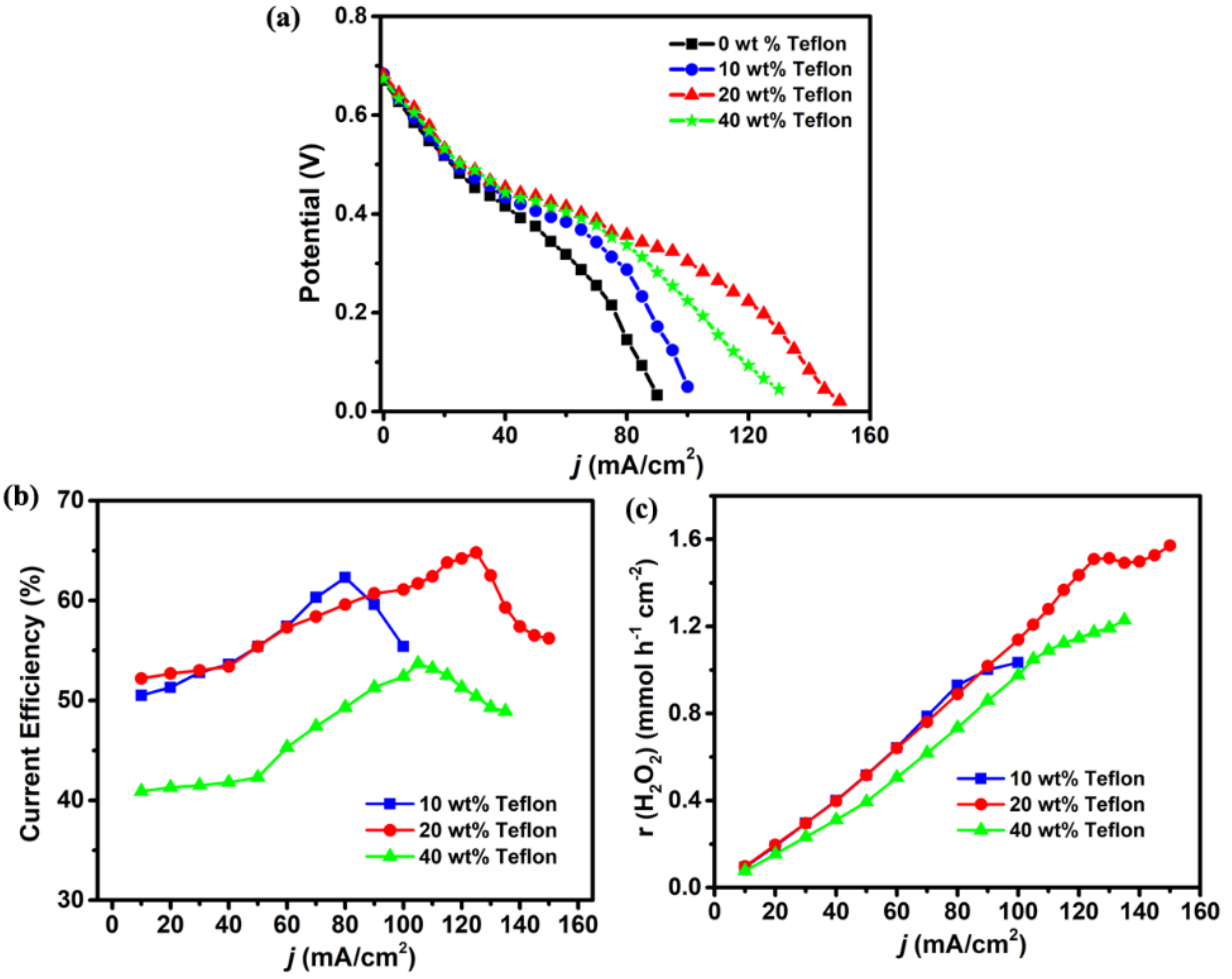

Supplementary Figure 33. Effect of Teflon content. (a) Polarization curves, (b) current efficiency, and (c) H_2O_2 production rate. Cathodic conditions: catalyst loading of 0.8 mg cm^{-2}, water flow rate of 5 mL min^{-1}, and fuel cell operation temperature of 80 °C.

Supplementary Figure 34

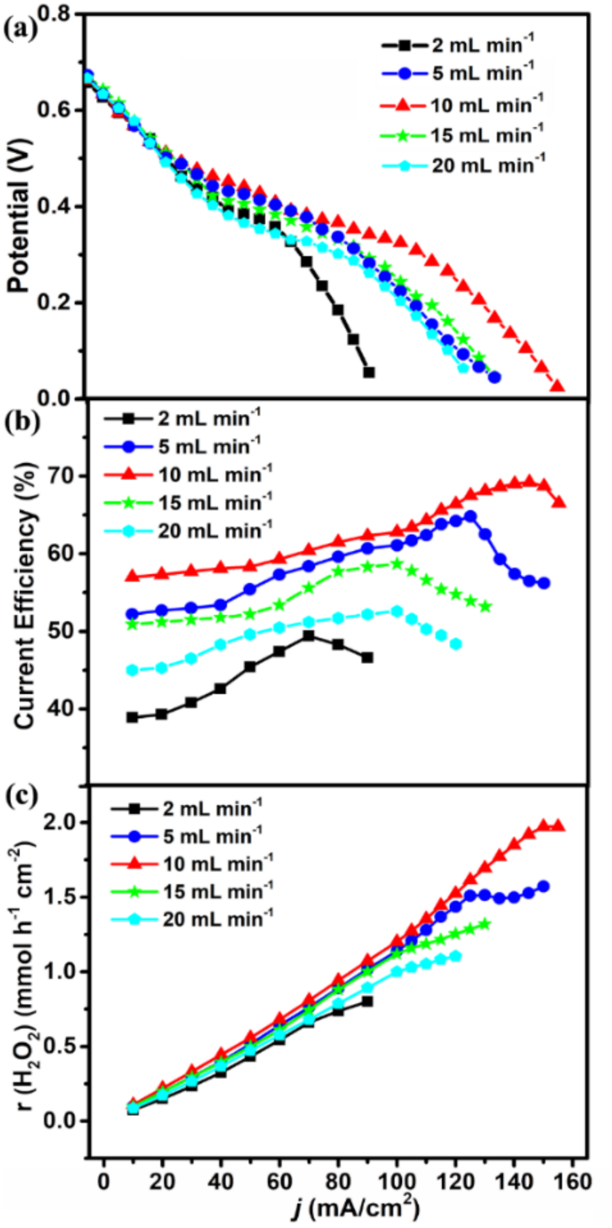

Supplementary Figure 34. Effect of water flow rate effect. (a) Polarization curves, (b) current efficiency, and (c) H_2O_2 production rate. Cathodic conditions: catalyst loading of 0.8 mg cm^{-2}, 20 wt% Teflon, and fuel cell operation temperature of 80 °C.

Supplementary Figure 35

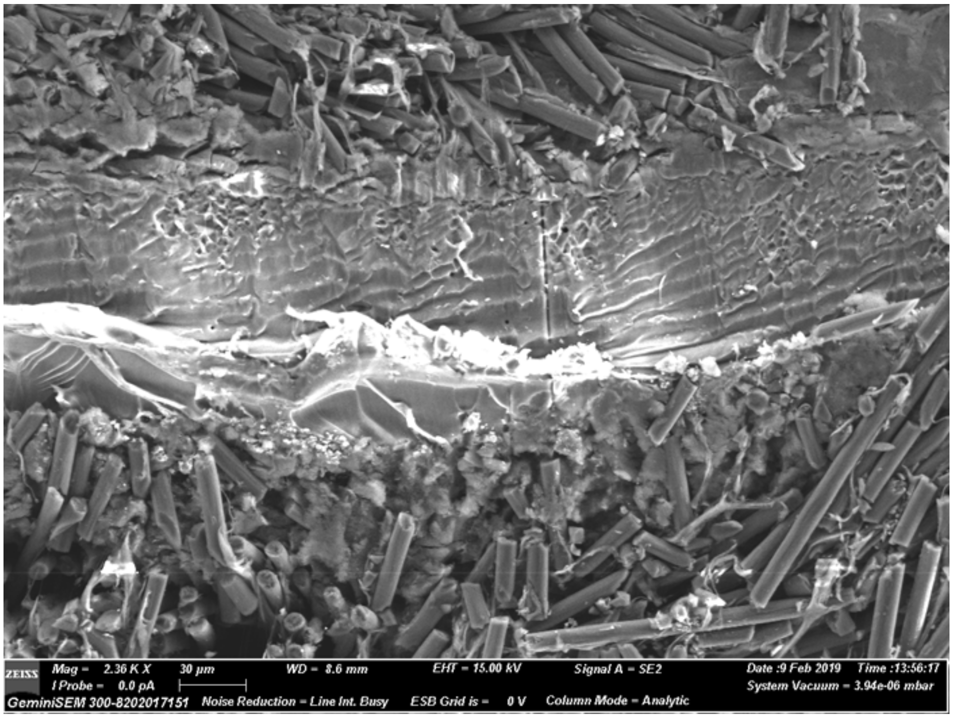

Supplementary Figure 35. Cross-sectional SEM image of the Pt/Nafion/Al_2O_3-PtP_2-600 MEA after 120 h at optimized conditions in H_2-O_2 fuel cell.

Supplementary Figure 36

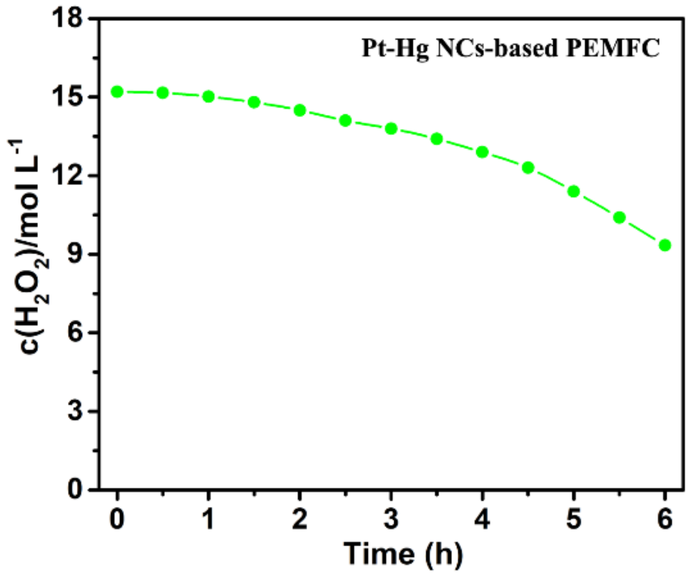

Supplementary Figure 36. Time-dependent neutral H_2O_2 concentration measured at a constant potential of 0.4 V for 6 h.

Supplementary Figure 37

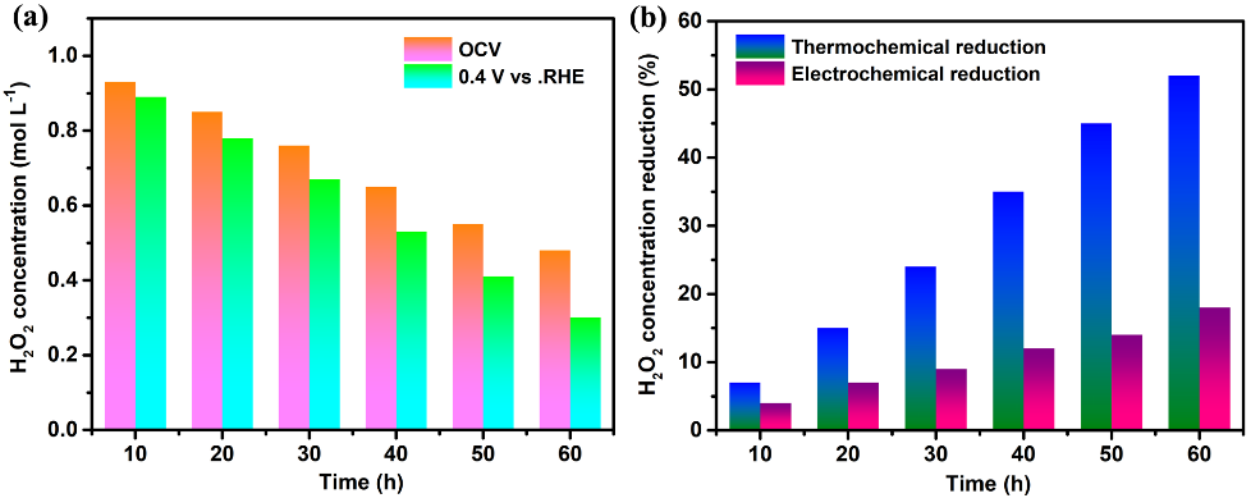

Supplementary Figure 37. Characterization of system under extended measurement. (a) Time-dependent H_2O_2 concentration measured at open-circuit voltage (OCV) and 0.4 V vs. RHE in an O_2-free 1 M H_2O_2 solution. (b) Time-dependent H_2O_2 concentration degradation from thermochemical and electrochemical reduction pathways.

Supplementary Figure 38

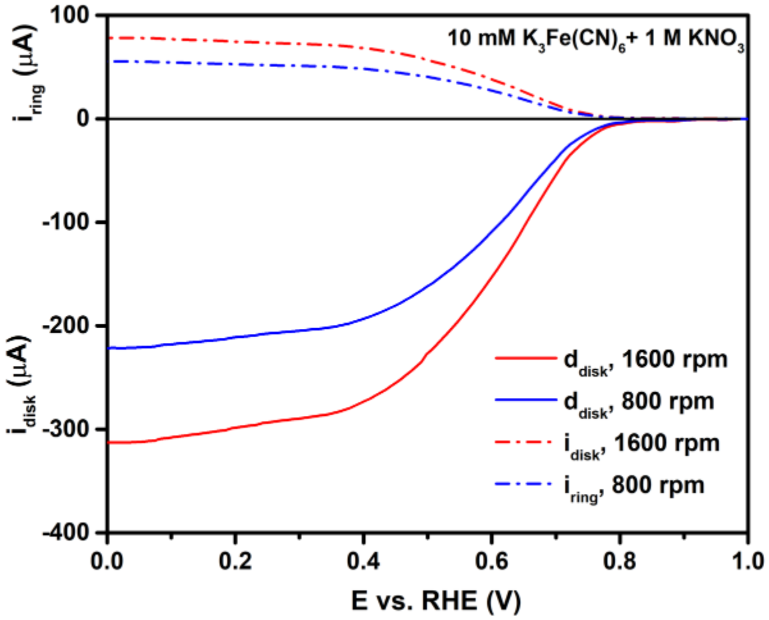

Supplementary Figure 38. LSV curves of the used RRDE in an O_2-saturated 0.1 M $HClO_4$ at a rotation speed of 800 and 1600 rpm (10 mV s^{-1}). The collection efficiency N is calculated to be 0.25, quite close to the theoretical value of old MTI34 RRDE from the Pine company.

Supplementary Table 1

Supplementary Table 1. Elemental composition determined by ICP-MS of Pt and P in the pristine and modified PtP_2 samples before and after ORR in acidic or neutral solution.

Sample	Weight% Pt	Weight% P	Atomic ratio Pt:P
PtP_2 (as synthesized)	74.3	23.7	0.498
PtP_2 (after acid-treatment)	73.7	23.2	0.504
PtP_2 (after ORR)	72.6	22.4	0.514
Al_2O_3/PtP_2-600 (as synthesized)	66.5	21.5	0.496
Al_2O_3/PtP_2-600 (after ORR)	65.8	21.3	0.491

Supplementary Table 2

Supplementary Table 2. Fitting results of Pt L_3-K edge EXAFS data. The coordination number (CN) and bond length obtained from the EXAFS spectra of the PtP_2 NCs and Pt NCs. Commercial PtO_2, $PtCl_2$, and Pt foil were used as references.

Sample	Bond	CN	R (Å)	σ^2 (10^{-3} Å^2)	R factor
PtP_2 NCs	Pt_1-P	7.2	2.20 ± 0.03	4.2	0.0082
PtP_2 NCs	Pt_2-P	6.6	2.85 ± 0.02	8.7	0.0082
Pt NCs	Pt-Pt	10.9	2.75 ± 0.02	5.8	0.0064
Pt NCs	Pt-Pt	7.5	2.89 ± 0.01	6.9	0.0064
PtO_2	Pt-O	6.3	1.65 ± 0.01	7.1	0.0062
PtO_2	Pt-Pt	7.2	2.71 ± 0.01	5.3	0.0062
$PtCl_2$	Pt-Cl	6.4	1.60 ± 0.02	4.9	0.0082
Pt foil	Pt-Pt	12.0	2.73 ± 0.01	4.0	0.0044

Supplementary Table 3

Table 3. Explanation of mass activity calculations for different electrocatalysts presented in Figure 2b of the main text.

Sample	Electrolyte	Loading (mg cm^{-2})	Comment on mass activity calculation	Ref.
Au-Pd	0.1 M $HClO_4$	0.020	For the optimized $Au_{0.7}Pd_{0.3}/C$, the generated H_2O_2 can be obtained by combining the ring current from Figure 5B with the mass loading and collection efficiency.	3
Pt-Hg	0.1 M $HClO_4$	0.014	The generated H_2O_2 can be obtained by the calculated peroxide current from Figure 4b and mass loading.	4
Pd-Hg	0.1 M $HClO_4$		The generated H_2O_2 can be obtained by the calculated peroxide current from Figure 3d and mass loading.	5
Pt/HSC	0.1 M $HClO_4$	0.050	The generated H_2O_2 can be obtained by H_2O_2 selectivity and polarization curve from Figure 3b and d. $N_{collection}$ = 0.2	6
Pt/TiN	0.1 M $HClO_4$	0.175	The generated H_2O_2 can be obtained by ring current from Figure 3a, collection efficiency, and mass loading.	7
Co-N-C	0.5 M H_2SO_4	0.100	The generated H_2O_2 can be obtained by ring current from Figure 2a. A_{disk} = 0.196 cm^2; $N_{collection}$ = 0.37.	8
Au-Pd	0.1 M $HClO_4$	0.010	The generated H_2O_2 can be obtained by ring current from Figure 3B, collection efficiency, and mass loading.	9
CoS_2	0.05 M H_2SO_4	0.305	The generated H_2O_2 can be obtained by ring current from Figure 2a A_{disk} = 0.126 cm^2; $N_{collection}$ = 0.43.	10
Co_1-NG(O)	0.1 M $HClO_4$	0.010	The generated H_2O_2 can be obtained by ring current from Figure 3f. A_{disk} = 0.2475 cm^2; $N_{collection}$ = 0.356.	11
h-Pt_1-CuS_x	0.1 M $HClO_4$	0.015	The generated H_2O_2 can be obtained by ring current from Figure 4a. A_{disk} = 0.2475 cm^2; $N_{collection}$ = 0.37.	12

Supplementary Table 4

Supplementary Table 4. ICP-MS concentrations in the solution after 6 h of ORR in 0.1 M $HClO_4$. The applied potential for RRDE is fixed at 0.5 V vs. RHE. The mass loading is 0.2 mg/cm^2. The background concentrations in the electrolyte was subtracted for all the reported values.

Samples	Volume of solution (mL)	[Pt] (ppb)	[P] (ppb)	[Hg] (ppb)
PtP_2	40	5.8	24.4	−
Pt-Hg	40	64.5	−	74.6×10^3

Supplementary Table 5

Supplementary Table 5. The absorption edge (E_0) and corresponding oxidation state (δ) of Pt in PtP_2 NCs under various applied potential (vs. RHE). PtO_2, $PtCl_2$, and Pt foil are used as references.

Samples	PtP_2 (Pristine)	PtP_2 (OCP)	PtP_2 (0.9 V)	PtP_2 (0.7 V)	PtP_2 (0.5 V)	PtP_2 (0.3 V)	PtO_2	$PtCl_2$	Pt foil
E_0 (eV)	11567.3	11567.4	11567.4	11566.7	11566.4	11566.4	11567.7	11566.2	11565.5
δ	3.27	3.29	3.29	2.72	2.25	2.25	4.00	2.00	0

Supplementary Table 6

Supplementary Table 6. Experimental variables for direct electrosynthesis of neutral H_2O_2 in a continuous H_2-O_2 fuel cell with 4 cm^2 geometric electrode area.

Property	Value	Current density ($mA\ cm^{-2}$)	H_2O_2 concentration ($mmol\ L^{-1}$)	Maximum current efficiency (%)	H_2O_2 production rate ($mmol\ h^{-1}\ cm^{-2}$)
Catalyst loading[1] ($mg\ cm^{-2}$)	4.0	50	5.37	43.2	0.41
Catalyst loading ($mg\ cm^{-2}$)	8.0	50	7.56	60.8	0.57
Catalyst loading ($mg\ cm^{-2}$)	16.0	20	3.12	62.7	0.24
Teflon treatment[2] (wt%)	10	80	12.40	62.3	0.93
Teflon treatment (wt%)	20	125	20.15	64.8	1.51
Teflon treatment (wt%)	40	105	14.03	53.7	1.05
Water flow rate[3] ($mL\ min^{-1}$)	2	70	21.88	49.4	0.66
Water flow rate ($mL\ min^{-1}$)	5	125	20.15	64.8	1.51
Water flow rate ($mL\ min^{-1}$)	10	145	12.48	69.2	1.92
Water flow rate ($mL\ min^{-1}$)	15	100	4.87	58.7	1.12
Water flow rate ($mL\ min^{-1}$)	20	100	3.27	52.6	1.00
Operating temperature[4] (°C)	RT	120	7.99	53.5	1.23
Operating temperature (°C)	40	150	14.70	78.8	2.26
Operating temperature (°C)	60	140	12.62	72.5	1.94
Operating temperature (°C)	80	145	12.48	69.2	1.92

[1] Cathode conditions: O_2 flow rate (150 mL min^{-1}), GDL Teflon content (0 wt%), water flow rate (5 mL min^{-1}), cell temperature (80 °C).

[2] Cathode conditions: O_2 flow rate (150 mL min^{-1}), catalyst mass loading (0.8 mg cm^{-2}), water flow rate (5 mL min^{-1}), cell temperature (80 °C).

[3] Cathode conditions: O_2 flow rate (150 mL min^{-1}), catalyst mass loading (0.8 mg cm^{-2}), GDL Teflon content (20 wt%), cell temperature (80 °C).

[4] Cathode conditions: O_2 flow rate (150 mL min^{-1}), catalyst mass loading (0.8 mg cm^{-2}), GDL Teflon content (20 wt%), water flow rate (10 mL min^{-1}).

The anode conditions for [1-4] are: H_2 flow rate (100 mL min^{-1}), Pt/C catalyst loading (1.0 mg_{Pt} cm^{-2}).

Supplementary Table 7

Supplementary Table 7. ICP-MS concentrations of the neutral catholyte solution in PtP_2 and Pt-Hg NCs-based PEMFC following non-recycle mode operation for 6 h. The applied potential is fixed at 0.4 V.

Cathodes in PEMFC	[Pt] (ppb)	[P] (ppb)	[Hg] (ppb)
PtP_2	5.8	11.7	−
Pt-Hg	15.8	−	5.9×10^3

Supplementary Methods

Computation details: First principles calculations were carried out using the Materials Studio with norm-conserving pseudopotentials and generalized gradient approximation (GGA) exchange-correlation functionals parameterized by Perdew-Burke-Enzerhof (PBE)[13]. A self-consistency convergence criterion of 10^{-6} eV was used for all calculations. All the structures were fully relaxed until force components on atoms were less than 10^{-3} eV/Å. The number of plane-waves was determined by a kinetic energy cutoff of 571.4 eV. Monkhorst-Pack grids with a maximum separation of 0.04 $Å^{-1}$ between k-points were used for sampling the Brillouin zone. This sampling density was checked with respect to the convergence of the bulk PtP_2 and Pt total energies, corresponding to a 4 × 4 × 1 k-point grid for the reciprocal space of the 12-atom PtP_2 primitive cell and the 4-atom Pt primitive cell. We studied free energy and binding energy of reaction intermediates adsorbed on PtP_2 (111) and Pt (111) surfaces with supercells separated by vacuum of 15 Å thickness. The slab thickness and the vacuum separation were chosen to converge the total energy of the systems into 10^{-3} eV.

Computation of free-energy for the ORR: We consider the associative mechanism with OOH*, O* and OH* as ORR intermediates for both the two (equation 1,2) and four (equation 1,3,4,5) electrons ORR reaction on PtP_2 and Pt in acidic solution.

$$* + O_2\,(g) + H^+ + e^- \rightarrow OOH* \quad (1)$$

$$OOH* + H^+ + e^- \rightarrow H_2O_2\,(l) + * \quad (2)$$

$$OOH* + H^+ + e^- \rightarrow O* + H_2O\,(l) \quad (3)$$

$$O* + H^+ + e^- \rightarrow OH* \quad (4)$$

$$OH* + H^+ + e^- \rightarrow H_2O\,(l) + * \quad (5)$$

where * and X* represent an adsorption site and an absorbed intermediate on the surface, respectively. To estimate the adsorption energy of different intermediates at zero potential and pH

= 0, we calculated the reaction energy of each individual intermediate and corrected the for zero-point energy (ZPE) and entropy (TS) using the equation: $\Delta G = \Delta E + \Delta ZPE - T\Delta S$. The entropy ($\Delta S$) and zero-point energy corrections (ΔZPE) were determined by employing the computed vibrational frequencies and standard tables for the reactants and products in the gas phase. Additionally, we used the computational hydrogen electrode (CHE) approach which exploits that the chemical potential of a proton-electron pair is equal to that of gas-phase H_2 at U = 0.0 V vs. the reversible hydrogen electrode (RHE). The effect of the electrode potential on the free energy of the intermediates is taken into account through shifting the electron energy by –eU where e and U are the elementary charge and the electrode potential, respectively[14]. A formation energy for the water molecule of -2.46 eV was adopted and a solvation energy (-0.22 eV) is applied to correct ΔE_{OH*} and ΔE_{OOH*}[14]. The reaction overpotential could be obtained by evaluating the difference between the reversible potential (1.23 V vs. RHE for 4e ORR or 0.70 V vs. RHE for 2e ORR) and the corresponding voltage needed for changing all the free-energy steps into downhill.

Supplementary References

1 Mazumder, V. & Sun, S. Oleylamine-mediated synthesis of Pd nanoparticles for catalytic Formic acid oxidation. *J. Am. Chem. Soc.* **131**, 4588-4589 (2009).

2 Mazumder, V. et al. A facile synthesis of MPd (M = Co, Cu) nanoparticles and their catalysis for formic acid oxidation. *Nano Lett.* **12**, 1102-1106 (2012).

3 Jirkovsky, J. S. et al. Single atom hot-spots at Au-Pd nanoalloys for electrocatalytic H_2O_2 production. *J. Am. Chem. Soc.* **133**, 19432-19441 (2011).

4 Siahrostami, S. et al. Enabling direct H_2O_2 production through rational electrocatalyst design. *Nat. Mater.* **12**, 1137-1143 (2013).

5 Verdaguer-Casadevall, A. et al. Trends in the electrochemical synthesis of H_2O_2: enhancing activity and selectivity by electrocatalytic site engineering. *Nano Lett.* **14**, 1603-1608 (2014).

6 Choi, C. H. et al. Tuning selectivity of electrochemical reactions by atomically dispersed platinum catalyst. *Nat. Commun.* **7**, 10922 (2016).

7 Yang, S., Kim, J., Tak, Y. J., Soon, A. & Lee, H. Single- atom catalyst of platinum supported on titanium nitride for selective electrochemical reactions. *Angew. Chem. Int. Ed.* **55**, 2058-2062 (2016).

8 Sun, Y. et al.Activity-selectivity trends in the electrochemical production of hydrogen peroxide over single-site metal-nitrogen-carbon catalysts. *J. Am. Chem. Soc.* **141**, 12372-12381 (2019).

9 Pizzutilo, E. et al. Gold–palladium bimetallic catalyst stability: consequences for hydrogen peroxide selectivity. *ACS Catal.* **7**, 5699-5705 (2017).

10 Sheng, H. et al. Electrocatalytic production of H_2O_2 by selective oxygen reduction using earth-abundant cobalt pyrite (CoS_2). *ACS Catal.* **9**, 8433-8442 (2019).

11 Jung. E. et al. Atomic-level tuning of Co-N-C catalyst for high-performance electrochemical H_2O_2 production. *Nat. Mater.* **19**, 436-442 (2020).

12 Shen, R. et al. High-concentration single atomic Pt sites on hollow CuSx for selective O_2 reduction to H_2O_2 in acid solution. *Chem* **5**, 2099-2110 (2019).

13 Perdew, J. P. Generalized gradient approximation made simple. *Phys. Rev. Lett.* **77**, 3865-3868 (1996).

14 Nørskov, J. K. Trends in the exchange current for hydrogen evolution. *J. Electrochem. Soc.* **152**, J23-J26 (2005).
